# SLC39A6 as a pan-cancer promising biomarker and actionable therapeutic target for CH5132799 sensitivity

**DOI:** 10.1186/s12885-026-16063-6

**Published:** 2026-05-18

**Authors:** Li Hongmin, Wang Yufei, Wang Yuwei, Yuan Dongqi, Li Mengjie, Su Yudong, Chen Peng, Zhang Jinghua

**Affiliations:** 1 Department of Thoracic Oncology, Lung Cancer Diagnosis and Treatment Center, Tianjin Medical University Cancer Institute and Hospital, Tianjin, 300060 China; 2 National Clinical Research Center for Cancer, State Key Laboratory of Druggability Evaluation and Systematic Translational Medicine, Tianjin’s Clinical Research Center for Cancer, Key Laboratory of Cancer Prevention and Therapy, Tianjin, 300060 China; 3 Department First of Oncology, Cangzhou Central Hospital, Cangzhou, 061000 China; 4 Department of Traumatic Orthopedics II, Cangzhou Central Hospital, Cangzhou, 061000 China; 5 Eighth Oncology Department, Tangshan People’s Hospital, Tangshan, 063000 China

**Keywords:** SLC39A6, Bioinformatics, Prognosis, Immunotherapy response, CH5132799

## Abstract

**Background:**

The zinc transporter SLC39A6, a member of the ZIP (Zrt-Irt-like protein) family, mediates zinc influx from the extracellular milieu into the cytosol and is indispensable for the function of numerous enzymes, transcription factors and signaling molecules. Previous studies have shown that SLC39A6 expression is associated with prognosis in esophageal squamous cell carcinoma and cervical cancer, indicating its potential impact on patient survival and tumor immunity. But a comprehensive pan-cancer analysis of SLC39A6 is still lacking. This study aimed to systematically delineate the oncogenic and prognostic relevance of SLC39A6 across multiple cancer types, to unravel its interplay with immune-infiltration patterns in the tumor micro-environment, and to preliminarily identify SLC39A6-associated therapeutic vulnerabilities.

**Methods:**

SLC39A6 expression profiles were retrieved from The Cancer Genome Atlas (TCGA) and cross-validated with GTEx, TIMER, HPA, cBioPortal, GEPIA2, STRING, KEGG, GO and other public repositories. Pan-cancer analyses were performed to characterize expression patterns, prognostic value, mutational landscape and functional networks. We further interrogated correlations between SLC39A6 and immune infiltration, tumor mutational burden (TMB), microsatellite instability (MSI) and immune-regulatory genes. Drug-sensitivity associations were evaluated using the CellMiner database, which facilitated molecular docking to predict binding poses and subsequent in vivo validation of lead compounds.

**Results:**

SLC39A6 exhibited marked dysregulation across diverse tumor types and was significantly linked to patient survival. High SLC39A6 expression is associated with reduced overall survival and progression-free survival, particularly in cervical squamous cell carcinoma (CESC), as evidenced by studies that have analyzed the gene's expression and its impact on patient survival. Immune deconvolution revealed robust associations between SLC39A6 levels and the abundance of cytotoxic T cells, dendritic cells, macrophages and other immune subsets. CellMiner analyses demonstrated that increased levels of SLC39A6 resulted in enhanced sensitivity to the PI3Kα inhibitor CH5132799. Molecular docking studies predicted a strong affinity between CH5132799 and the zinc-binding pocket of SLC39A6, while mouse xenograft models further validated that CH5132799 effectively inhibited SLC39A6-mediated tumor growth.

**Conclusion:**

SLC39A6 regulates the dynamics of immune infiltration and impacts prognosis in a wide range of malignancies. It emerges as a promising biomarker for prognosis, immunology, and therapy in the field of precision oncology.

**Supplementary Information:**

The online version contains supplementary material available at 10.1186/s12885-026-16063-6.

## Introduction

Cancer continues to be one of the primary causes of illness and death globally. Breast invasive carcinoma, cholangiocarcinoma, colon adenocarcinoma, and lung adenocarcinoma together contribute to millions of new cases and deaths each year, exerting significant clinical and socioeconomic pressures [1]. Despite recent progress in surgery, cytotoxic chemotherapy, and radiotherapy, the effectiveness of treatments remains limited and frequently comes with severe side effects [2]. Consequently, there is an urgent need for reliable molecular biomarkers to guide personalized therapy.

The solute carrier family 39 member 6 (SLC39A6, also known as ZIP6) encodes a zinc transporter that regulates intracellular zinc homeostasis [3]. Dysregulated zinc signaling has been causally linked to tumorigenesis and malignant progression [4]. Over the past five years, numerous studies have documented that SLC39A6 is frequently up-regulated in breast, colorectal, and lung cancers, where higher expression correlates with advanced stage and shorter overall survival [5–7]. These findings support the candidacy of SLC39A6 as a pan-cancer prognostic marker.

Beyond prognosis, the therapeutic implications of SLC39A6 remain underexplored. We conducted an integrative drug sensitivity analysis to identify cytotoxic and targeted agents whose efficacy is significantly associated with SLC39A6 expression. This in-silico drug-sensitivity screen has nominated candidate compounds that may preferentially benefit SLC39A6-high tumors and provides a mechanistic rationale for repositioning existing drugs, thereby accelerating translational applications.

Recent pan-cancer studies have explored various molecular markers and analytical tools, providing valuable paradigms and methodologies for investigating potential cancer biomarkers and therapeutic targets, such as the research on ZNF165 as a pan-cancer prognostic biomarker and the development of the DrugSurvPlot platform for drug sensitivity and survival analysis. These studies, along with single-cell pan-cancer analyses and integrated analyses of cancer-related genes, offer important references for systematically exploring the role of SLC39A6 in pan-cancer progression and its potential as a therapeutic target [8–11].To systematically characterize SLC39A6 across malignancies, we conducted a comprehensive pan-cancer study using The Cancer Genome Atlas (TCGA) dataset accessed via TCGA biolinks [12]. We employed Cox regression, Kaplan–Meier analyses, immune-infiltration profiling, and drug-sensitivity modeling to delineate the associations among SLC39A6 expression, clinical outcomes, and therapeutic response. The overarching goal is to clarify the expression patterns of SLC39A6 across cancer types, to validate its prognostic value, and to nominate druggable vulnerabilities for SLC39A6-driven tumors.

## Materials and methods

### Data download

By R package TCGA biolinks from Cancer Genome project (The Cancer Genome Atlas, TCGA) (https://portal.gdc.cancer.gov/) to download 33 Cancer data set, The expression of matrix, respectively is: Adrenocortical carcinoma (ACC), Bladder urothelial carcinoma (BLCA), Breast invasive carcinoma (BRCA), Cervical endocervical adenocarcinoma and squamous cell carcinoma (CESC), Cholangiocarcinoma (CHOL), Colon adenocarcinoma (COAD), Lymphoid neoplasm diffuse large b-cell lymphoma (DLBC), Esophageal carcinoma (ESCA), Glioblastoma multiforme (GBM), Head and neck squamous cell carcinoma(HNSC), Kidney chromophobe (KICH), Kidney renal clear cell carcinoma (KIRC), Kidney renal papillary cell carcinoma (KIRP), Acute myeloid leukemia (LAML), Brain lower grade glioma (LGG), Liver hepatocellular carcinoma (LIHC), Lung adenocarcinoma (LUAD), Lung squamous cell carcinoma (LUSC), Mesothelioma (MESO), Ovarian serous cystadenocarcinoma (OV), Prostate adenocarcinoma (PAAD), Pheochromocytoma and paraganglioma (PCPG), Pancreatic adenocarcinoma (PRAD), Rectum adenocarcinoma (READ), Sarcoma (SARC), Skin cutaneous melanoma (SKCM), Stomach adenocarcinoma, (STAD), Testicular germ cell tumors (TGCT), Thyroid carcinoma (THCA), Thymoma (THYM), Uterine corpus endometrial carcinoma (UCEC), Uterine carcinosarcoma (UCS). All Tumor samples and Normal samples in the above 33 TCGA cancer types dataset were included in the analysis study. The gene expression of each sample was standardized to FPKM (Fragments Per Kilobase Million) format. The corresponding clinical data were obtained through the UCSC Xena database [13] (https://xena.ucsc.edu/).

### Comparative analysis of SLC39A6 pan-cancer level tumors and normal groups

Based on the sample grouping of 33 TCGA cancer datasets, samples were categorized into tumor and normal groups. Subsequently, the Wilcoxon Rank Sum Test was utilized to analyze the expression differences of SLC39A6 across the 33 TCGA cancer datasets. The R package ggplot2 (version 3.4.4) was employed to generate box plots illustrating the expression levels of SLC39A6 in both the tumor and normal groups of the 33 TCGA cancer datasets. Additionally, group comparison plots were created to highlight the differences between the two groups.

### Comparison of SLC39A6 between pan-cancer level pathological groups

To investigate the relationship between the expression of the single-gene SLC39A6 and the pathological stage of cancer, the differences in SLC39A6 expression across various pathological stages were analyzed. The expression profiles of SLC39A6 from 33 TCGA cancer datasets were categorized into normal, Stage I, Stage II, Stage III, and Stage IV groups. A comparative group diagram was then created to illustrate the analysis findings.

### Pan-cancer level prognostic analysis of SLC39A6

To evaluate the prognostic value of the SLC39A6 gene across 33 TCGA cancer types, a univariate Cox regression analysis was conducted using the R package 'survival((version 3.5–7). This analysis aimed to assess the impact of SLC39A6 on prognosis and to determine if it served as an independent prognostic factor [14]. In the univariate Cox regression analysis, the prognostic value of SLC39A6 was characterized by a cancer category with a *p*-value less than 0.05. A pan-cancer univariate Cox regression model was constructed, and the grouped expression levels of SLC39A6 in each cancer type were visualized using a Forest Plot.

For cancer types with a *P*-value < 0.05 for SLC39A6, Kaplan–Meier curve analysis was conducted using the R package survival (Version 3.5–7), and a KM curve was plotted based on the expression level of SLC39A6 [14, 15]. Overall survival (OS), Disease-Specific Survival (DSS), and Progression-Free Interval (PFI) were compared between groups with high and low SLC39A6 expression.

Finally, in the prognostic analysis of overall survival (OS), disease-specific survival (DSS), and progression-free interval (PFI), the key cancer types with a *p*-value less than 0.05 were intersected to identify the significant cancer types.

### Pan-cancer ssGSEA immune infiltration analysis

Single-Sample Gene-Set Enrichment Analysis (ssGSEA), also known as single-sample gene-set enrichment analysis, quantifies the relative abundance of each immune cell infiltrate [16]. Initially, immune cell types such as ADCs, B cells, CD8 T cells, and other human immune cell subtypes were labeled. Then, the enrichment scores calculated by ssGSEA analysis were used to represent the relative abundance of each immune cell type in each sample, resulting in an immune infiltration matrix for 33 TCGA cancer types. Next, the correlation between SLC39A6 and the abundance of immune cell infiltration was calculated using the Spearman algorithm, and the R package ggplot2 (Version 3.3.4) was employed to create a correlation heat map to display the results of the correlation analysis. For the key cancer types, the R package ggplot2 (Version 3.4.4) was used to generate group comparison maps, illustrating the expression differences of immune cells between the high and low expression groups of SLC39A6 in these cancer types.

### Correlation analysis of pan-cancer immune checkpoint gene (ICG) and mismatch repair (MMR) genes

Immune Checkpoint Genes (ICG) refer to ligand-receptor pairs that inhibit or stimulate immune responses. Immune checkpoint plays a crucial immunomodulatory role in maintaining immune homeostasis and preventing autoimmunity. Immune checkpoint blockade therapy has brought significant clinical benefits against several solid malignancies. Studies have published on the PubMed website (https://pubmed.ncbi.nlm.nih.gov/) literature that includes 47 (ICG) on immune checkpoint genes [17]. Subsequently, heat maps of the correlation between pan-cancer immune checkpoint genes (ICG) and SLC39A6 were drawn to display the results of correlation analysis across 33 TCGA cancer types.

Mismatch Repair (MMR) refers to the repair of DNA molecules containing mismatched bases to restore the nucleotide sequence to normal. The PubMed website (https://pubmed.ncbi.nlm.nih.gov/) has published literature indicating the presence of five MMR genes: MLH1, MSH2, MSH6, PMS2, and EPCAM. Associations between SLC39A6 and MMR genes were evaluated, and heatmaps were constructed [18].

### Genetic variant analysis

CBioPortal database(https://www.cbioportal.org/) is a collection of search, download, analysis, and visualization of cancer genomics data in a database, the integration of genomic data type is very broad,The database includes somatic mutations, DNA copy number alterations (CNAs), mRNA and microRNA (miRNA) expression, DNA methylation, protein abundance, and phosphoprotein abundance [19–21]. CBioPortal can perform a variety of analyses, but the main ones are mutation-related analyses and their visualization. In addition, cBioPortal database not only supports the query of single gene and single cancer, but also can conduct the analysis of multi-gene single cancer, single-gene multiple cancer, multi-gene multiple cancer, and even cross-cancer genome projects. The differences of Tumor Mutation Burden (TMB) and Microsatellite Instability (MSI) of SLC39A6 gene in 33 TCGA cancer types were analyzed by cBioPortal database.

### PPI interaction network and Gene ontology (GO) enrichment analysis

Protein–Protein Interaction Network (PPI) is composed of proteins and proteins that interact with each other and participate in all aspects of life processes such as biological signal transmission, gene expression regulation, energy and substance metabolism, and cell cycle regulation. Systematic analysis of protein–protein interactions in biological systems is of great significance for understanding the working principle of proteins in biological systems, understanding the reaction mechanism of biological signals and energy and substance metabolism under special physiological conditions such as diseases, and understanding the functional relationship between proteins. The STRING database (https://string-db.org/) is a database for searching the interactions between known and predicted proteins [22]. In this study, the STRING database was applied based on SLC39A6 gene with a minimum required interaction score greater than 0.400,and medium confidence (0.400) was used to construct the SLC39A6 gene-related PPI interaction network. The tightly connected local regions in the PPI interaction network may represent molecular complexes with specific biological functions. Interacting Genes in PPI network were selected for subsequent analysis.

Gene Ontology (GO) analysis is a common method for conducting large-scale functional enrichment studies, including Biological Process (BP), Cell Component (CC), and Molecular Function (MF) [23]. We used the R package clusterProfiler to perform gene ontology (GO) enrichment analysis of SLC39A6 gene and Interacting Genes [24]. The entry screening criteria were adj.p < 0.05 and FDR value (q value) < 0.25. The *p* value correction method was Benjamini-Hochberg (BH).

### Drug sensitivity of SLC39A6 in pan-cancer

To analyze the drug sensitivity of SLC39A6 in pan-cancer, CellMiner™ was used to obtain NCI-60 compound activity data and RNA-seq expression profiles [25, 26]. Analysis using R version 4.4.1 to selecte FDA-approved or clinical trial drugs. The structures of drugs and key target proteins were obtained from the PubChem database (https://pubchem.ncbi.nlm.nih.gov) and the PDB database (https://www.rcsb.org). The protein structures were dehydrated using PyMOL 3.0 software. Proteins and ligands were processed with AutoDockTools 1.5.7, followed by molecular docking using AutoDock Vina 1.1.2. Finally, chemical bond analysis of the results was performed using LigPlot + software, and visualization was conducted with PyMOL 3.0.

### Xenograft models

All experimental procedures were performed in accordance with relevant institutional and national guidelines and approved by the Institutional Animal Care and Use Committee of the Tianjin XINRUI (permit no. XinRui-DWLL-2025012). A total of 5 × 10^6^ A549 cells that had been suspended in 150 μL serum-free culture medium were injected subcutaneously into the left flanks of female BALB/c nude mice (4 weeks old, 15 ± 3 g weight, SPF (Beijing)Biotechnology CO.,Ltd).Tumor volume (V) was calculated with the following formula: length × width^2^ × 1/2. The mice were randomly divided into three groups (*n* = 4 or 5 per group). (1) Control group: mice were injected with A549 SCL39A6-vector. (2) SLC39A6 over expression group: mice were injected with A549 SCL39A6-OE. (3) CH5132799 group: mice were injected with A549 SCL39A6-OE and CH5132799 was orally administered at 12.5 mg/kg once a day.The animals were euthanized under deep anesthesia via cervical dislocation. Anesthesia was induced by intraperitoneal injection of a combination of ketamine (100 mg/kg) and xylazine (10 mg/kg). The humane endpoint was a tumor volume of 2000 mm^3^ or a diameter of 15 mm, whichever was reached first.

### Statistical analysis

**Fig. 1:**
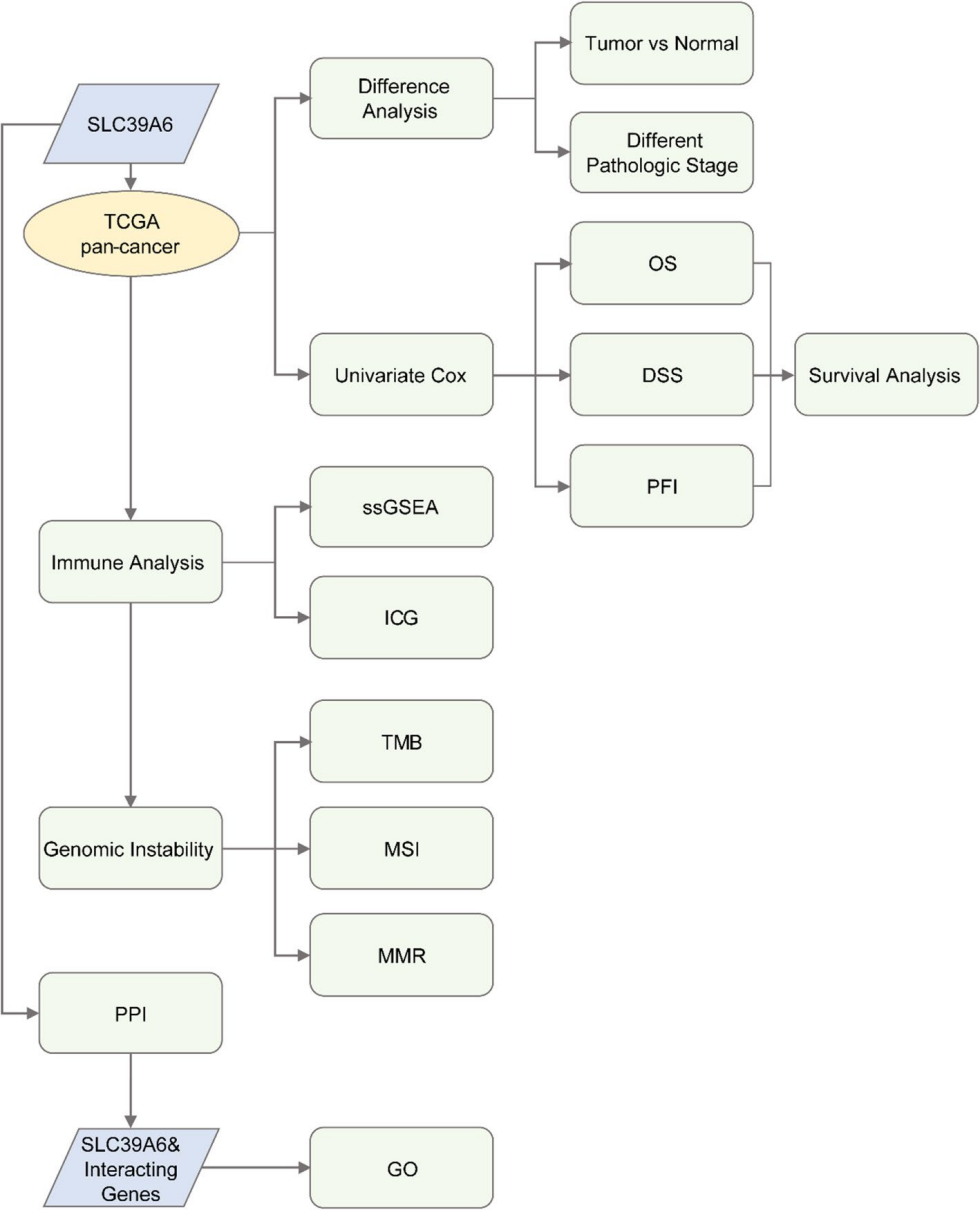
Flow chart for the comprehensive analysis of SLC39A6. TCGA, The Cancer Genome Atlas; PPI, Protein–Protein Interaction; ssGSEA, single-sample Gene-Set Enrichment Analysis; ICG, Immune Checkpoint Genes; TMB, Tumor Mutation Burden; MSI, Microsatellite Instability; MMR, Mismatch Repair; GO, Gene Ontology; OS, Overall survival; DSS, Disease Specific Survival; PFI, Progress Free Interval

All data processing and analysis in this article were based on R software (Version 4.2.2). If not otherwise specified, statistical significance of normally distributed variables was estimated by independent Student's T-Test for comparisons of continuous variables between two groups. Mann–Whitney U Test (Wilcoxon Rank Sum Test) was used to analyze the differences between variables that were not normally distributed. Kruskal–Wallis test was used for comparison of three or more groups. Spearman correlation analysis was used to calculate the correlation coefficient between different molecules. All statistical p values were two-sided if not specified, and a p value of less than 0.05 was considered to indicate statistical significance Fig. 1.

## Results

### Technology roadmap

#### Comparison of SLC39A6 pan-cancer level tumors and normal groups

**Fig. 2:**
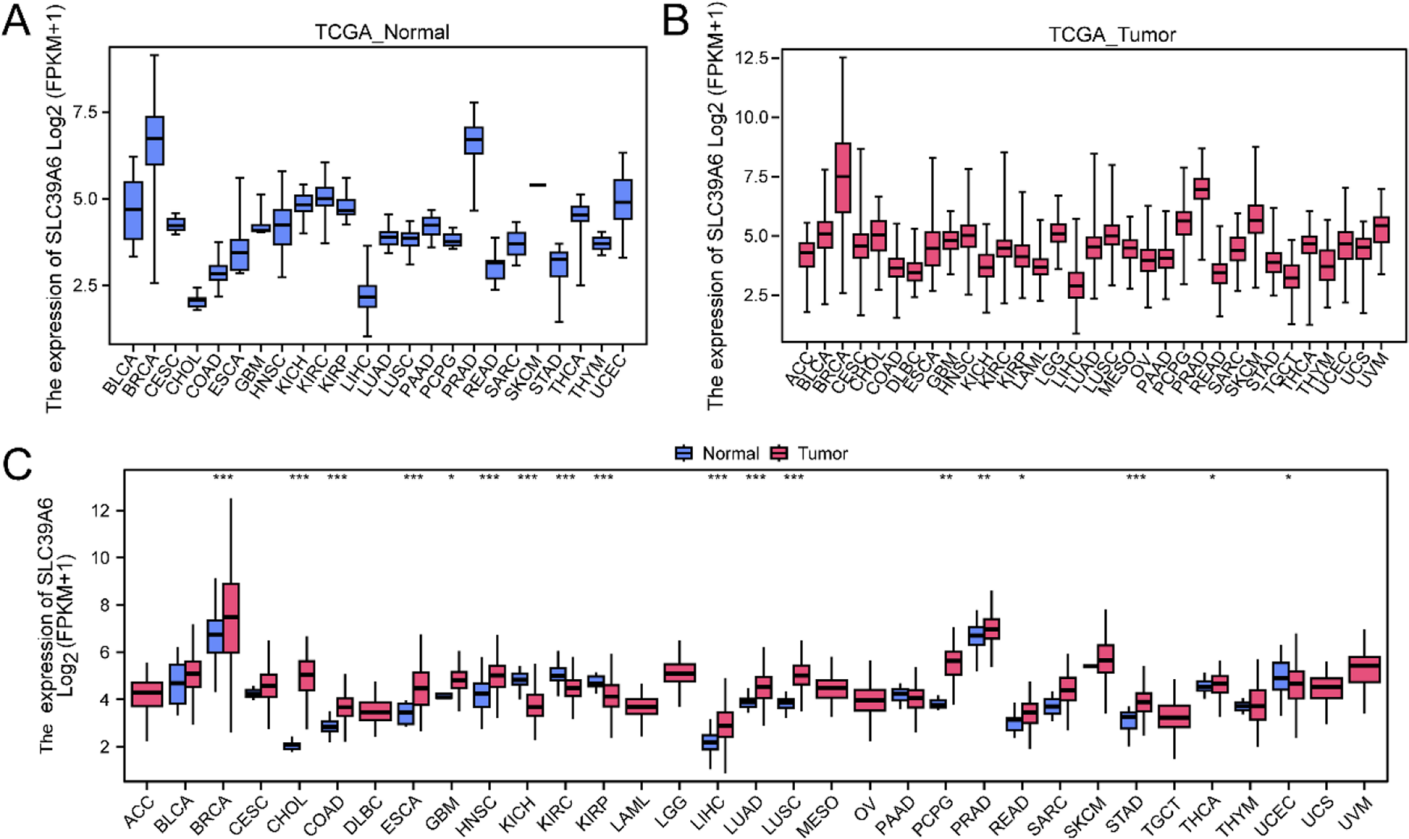
Pan-Cancer Expression Analysis of SLC39A6. **A** Box plot of pan-cancer expression of SLC39A6 gene in the normal (Normol) group. **B** Box plot of pan-cancer expression of SLC39A6 gene in Tumor group. **C** Box plot of pan-cancer expression group comparison of SLC39A6 gene. TCGA, The Cancer Genome Atlas; ACC, Adrenocortical carcinoma; BLCA, Bladder urothelial carcinoma; BRCA, Breast invasive carcinoma; CESC, Cervical endocervical adenocarcinoma and squamous cell carcinoma; CHOL, Cholangiocarcinoma; COAD, Colon adenocarcinoma; DLBC, Lymphoid neoplasm diffuse large b-cell lymphoma; ESCA, Esophageal carcinoma; GBM, Glioblastoma multiforme; HNSC, Head and neck squamous cell carcinoma; KICH, Kidney chromophobe; KIRC, Kidney renal clear cell carcinoma; KIRP, Kidney renal papillary cell carcinoma; LAML, Acute myeloid leukemia; LGG, Brain lower grade glioma; LIHC, Liver hepatocellular carcinoma; LUAD, Lung adenocarcinoma; LUSC, Lung squamous cell carcinoma; MESO, Mesothelioma; OV, Ovarian serous cystadenocarcinoma; PAAD, Prostate adenocarcinoma; PCPG, Pheochromocytoma and paraganglioma; PRAD, Pancreatic adenocarcinoma; READ, Rectum adenocarcinoma; SARC, Sarcoma; SKCM, Skin cutaneous melanoma; STAD, Stomach adenocarcinoma; TGCT, Testicular germ cell tumors; THCA, Thyroid carcinoma; THYM, Thymoma; UCEC, Uterine corpus endometrial carcinoma; UCS, Uterine carcinosarcoma; UVM, Uveal melanoma. The blue group is the Normal sample, and the red group is the Tumor sample. * represents *p* value < 0.05, which is statistically significant; ** represents *p* value < 0.01, highly statistically significant; *** represents *p* value < 0.001 and highly statistically significant

In order to explore the differential expression of SLC39A6 in 33 TCGA cancers, the expression and differential analysis results of SLC39A6 in the Tumor group and the normal group in 33 TCGA cancers were displayed by box plots (Fig. 2A-B) and group comparison plots (Fig. 2C). Differential results showed, SLC39A6 in breast invasive carcinoma (BRCA), cholangiocarcinoma (CHOL), colon cancer (COAD), Esophageal cancer (ESCA), glioblastoma multiforme (GBM), squamous cell carcinoma of head and neck (HNSC), chromophobe renal cell carcinoma (KICH), clear cell renal cell carcinoma (KIRC), squamous cell carcinoma of head and neck (HNSC), renal cell carcinoma of chromophobe (KICH), renal cell carcinoma of clear cell (KIRC). Renal papillary cell carcinoma (KIRP), Hepatocellular carcinoma (LIHC), Lung adenocarcinoma (LUAD), Lung squamous cell carcinoma (LUSC), Pheochromocytoma and paraganglioma (PCPG), Prostate cancer (PRAD), Rectal adenocarcinoma (READ), gastric adenocarcinoma (STAD), Thyroid carcinoma (THCA).There was a statistically significant difference in the expression level between the tumor group and the normal group of endometrial carcinoma (UCEC) (*p* < 0.05).

### Comparative analysis of SLC39A6 pan-cancer level pathological groups

**Fig. 3:**
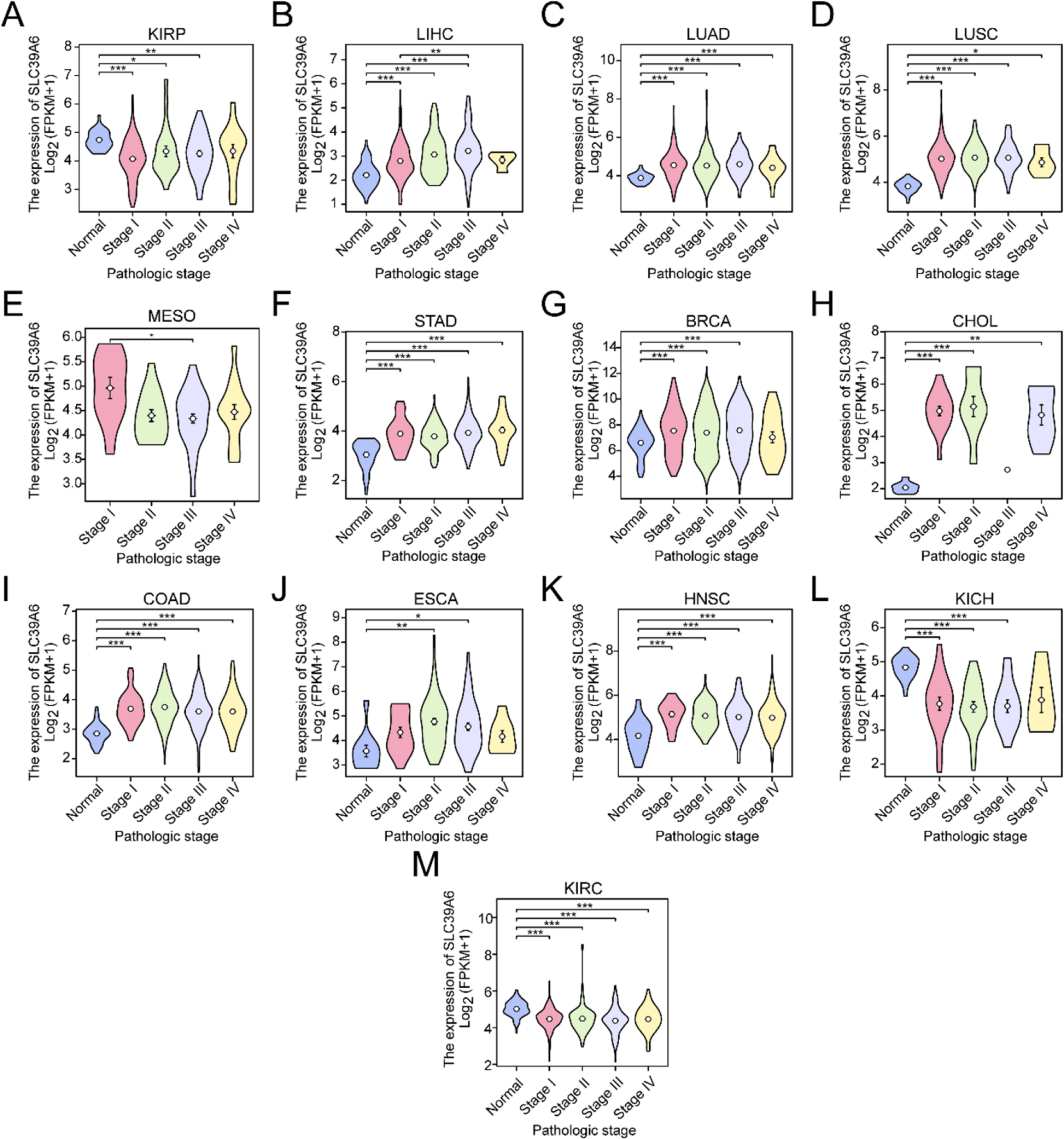
Difference analysis of SLC39A6 between pathologic stages. **A** m SLC39A6 gene in renal papillary carcinoma (KIRP) (**A**), hepatocellular carcinoma (LIHC) (**B**), lung adenocarcinoma (LUAD) (**C**), lung squamous carcinoma (LUSC) (**D**), mesothelioma (MESO) (**E**), gastric adenocarcinoma (STAD) (**F**), mammary ductal carcinoma (BRCA) (**G**), Violin plot of group comparison between different cancer pathological stages of cholangiocarcinoma (CHOL) (**H**), colon cancer (COAD) (**I**), esophageal cancer (ESCA) (**J**), head and neck squamous cell carcinoma (HNSC) (**K**), renal chromophobe cell carcinoma (KICH) (**L**), renal clear cell carcinoma (KIRC) (**M**). TCGA, The Cancer Genome Atlas; BRCA, Breast invasive carcinoma; CHOL, Cholangiocarcinoma; COAD, Colon adenocarcinoma; ESCA, Esophageal carcinoma; HNSC, Head and neck squamous cell carcinoma; KICH, Kidney chromophobe; KIRC, Kidney renal clear cell carcinoma; KIRP, Kidney renal papillary cell carcinoma; LIHC, Liver hepatocellular carcinoma; LUAD, Lung adenocarcinoma; LUSC, Lung squamous cell carcinoma; MESO, Mesothelioma; STAD, Stomach adenocarcinoma. * represents *p* value < 0.05, statistically significant; ** represents *p* value < 0.01, highly statistically significant; *** represents *p* value < 0.001 and highly statistically significant. Blue is Normal samples, pink is Stage Ⅰ group, green is Stage ⅠⅠ group, purple is Stage ⅠⅠⅠ group, and yellow is Stage Ⅳ group

In order to explore the relationship between the expression of single-gene SLC39A6 and the pathological stage of cancer, the difference of the expression of single-gene SLC39A6 in different pathological stages of cancer was analyzed. The group comparison figure (Fig. 3A-M) shows the difference analysis results between different pathological stages of SLC39A6 in TCGA cancer types with significant differences. Differential results showed (Fig. 3A-M), SLC39A6 in breast invasive carcinoma (BRCA), cholangiocarcinoma (CHOL), colon cancer (COAD), Esophageal cancer (ESCA), squamous cell carcinoma of head and neck (HNSC), Chromophobe renal cell carcinoma (KICH), clear cell renal cell carcinoma (KIRC), papillary renal cell carcinoma (KIRP), squamous cell carcinoma of head and neck (HNSC). The expression levels of hepatocellular carcinoma (LIHC), lung adenocarcinoma (LUAD), lung squamous cell carcinoma (LUSC), mesothelioma (MESO), and gastric adenocarcinoma (STAD) were statistically significant between different cancer pathological stages (*p* < 0.05).

### Pan-cancer level prognostic analysis of SLC39A6

**Fig. 4:**
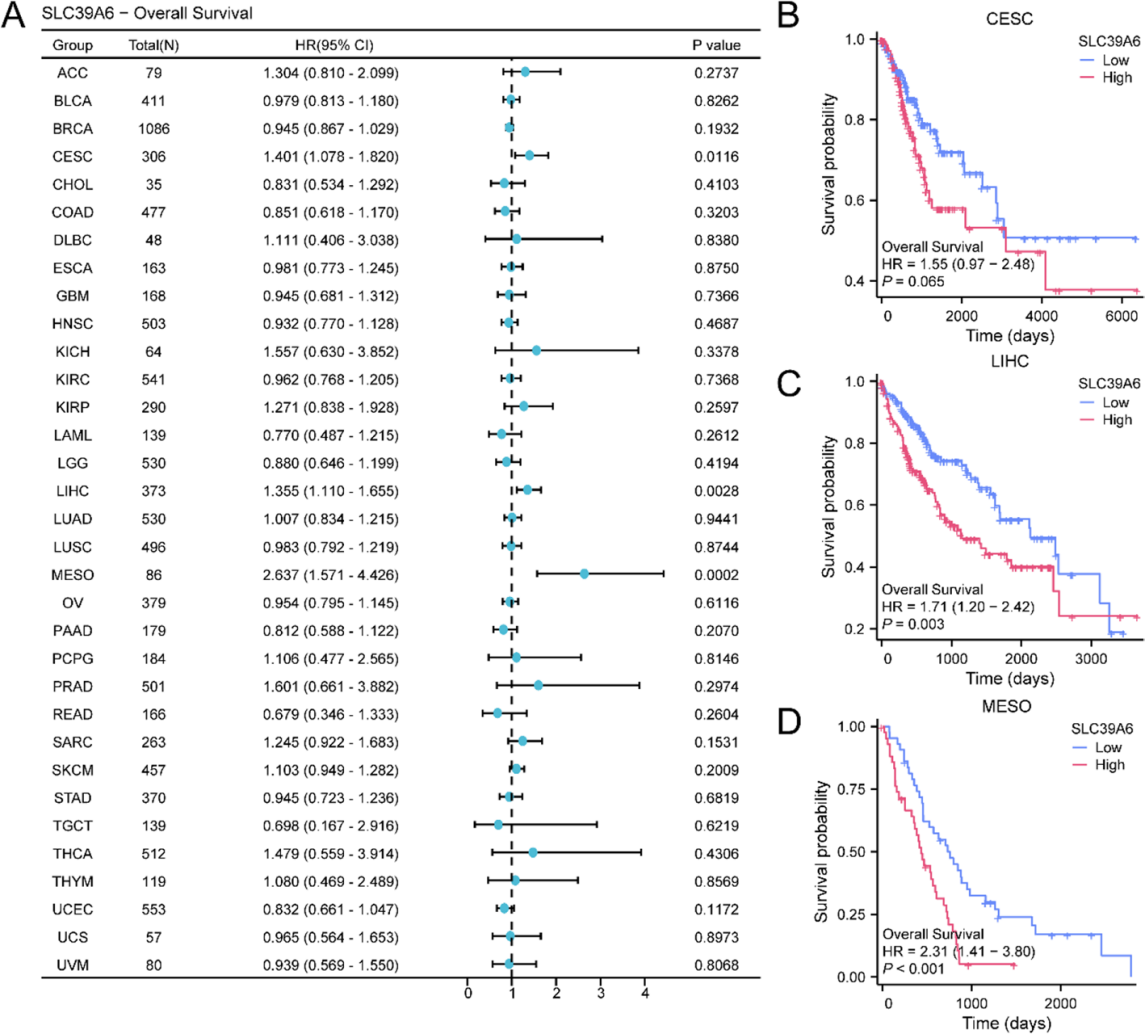
Association between SLC39A6 Expression and Overall Survival (OS). **A** Forest plot of the results of single-gene Cox analysis of SLC39A6 overall survival (OS) in cancer samples from the TCGA dataset. **B**-**D** Kaplan–meier (KM) curves of SLC39A6 in cervical squamous cell carcinoma and adenocarcinoma (CESC) (**B**), hepatocellular carcinoma (LIHC) (**C**), and mesothelioma (MESO) (**D**) in cancer samples from TCGA dataset. TCGA, The Cancer Genome Atlas; ACC, Adrenocortical carcinoma; BLCA, Bladder urothelial carcinoma; BRCA, Breast invasive carcinoma; CESC, Cervical endocervical adenocarcinoma and squamous cell carcinoma; CHOL, Cholangiocarcinoma; COAD, Colon adenocarcinoma; DLBC, Lymphoid neoplasm diffuse large b-cell lymphoma; ESCA, Esophageal carcinoma; GBM, Glioblastoma multiforme; HNSC, Head and neck squamous cell carcinoma; KICH, Kidney chromophobe; KIRC, Kidney renal clear cell carcinoma; KIRP, Kidney renal papillary cell carcinoma; LAML, Acute myeloid leukemia; LGG, Brain lower grade glioma; LIHC, Liver hepatocellular carcinoma; LUAD, Lung adenocarcinoma; LUSC, Lung squamous cell carcinoma; MESO, Mesothelioma; OV, Ovarian serous cystadenocarcinoma; PAAD, Prostate adenocarcinoma; PCPG, Pheochromocytoma and paraganglioma; PRAD, Pancreatic adenocarcinoma; READ, Rectum adenocarcinoma; SARC, Sarcoma; SKCM, Skin cutaneous melanoma; STAD, Stomach adenocarcinoma; TGCT, Testicular germ cell tumors; THCA, Thyroid carcinoma; THYM, Thymoma; UCEC, Uterine corpus endometrial carcinoma; UCS, Uterine carcinosarcoma; UVM, Uveal melanoma; OS, Overall Survival; HR, Hazard Ratio; CI, Confidence Intervals; KM, Kaplan–Meier. The SLC39A6 Low expression group (Low) is blue, and the SLC39A6 High expression group (High) is pink

A survival analysis of SLC39A6 expression in each cancer was performed based on TCGA dataset cancer samples, and the positive results are shown in Table 1. First, the results of single-gene Cox analysis of SLC39A6 overall survival (OS) showed that SLC39A6 was significant in the single-gene Cox model of cervical squamous cell carcinoma and adenocarcinoma (CESC), hepatocellular carcinoma (LIHC), and mesothelioma (MESO) (*p* value < 0.05). The Kaplan–Meier (KM) curves of SLC39A6 in the overall survival (OS) of the above three cancers are shown (Fig. 4B-D), hepatocellular carcinoma (LIHC), The overall survival (OS) of mesothelioma (MESO) was significantly different between high and low SLC39A6 expression groups (*p* < 0.05).

**Table 1:** Results of univariate cox

Tumor	Outcome	Total(N)	HR(95% CI)	*P* value
CESC	OS	306	1.401 (1.078–1.820)	0.0116
LIHC	OS	373	1.355 (1.110–1.655)	0.0028
MESO	OS	86	2.637 (1.571–4.426)	0.0002
BRCA	DSS	1066	0.854 (0.757–0.963)	0.0098
LIHC	DSS	365	1.344 (1.038–1.740)	0.0249
MESO	DSS	66	3.442 (1.758–6.740)	0.0003
ACC	PFI	79	1.748 (1.150–2.658)	0.0090
BRCA	PFI	1086	0.905 (0.828–0.990)	0.0286
CESC	PFI	306	1.544 (1.174–2.030)	0.0019
LIHC	PFI	373	1.235 (1.037–1.470)	0.0178
MESO	PFI	84	1.882 (1.082–3.275)	0.0253

*ACC* Adrenocortical carcinoma, *BRCA* Breast invasive carcinoma, *CESC* Cervical endocervical adenocarcinoma and squamous cell carcinoma, *LIHC* Liver hepatocellular carcinoma, *MESO* Mesothelioma, *OS* Overall Survival, *DSS* Disease Specific Survival, *PFI* Progress Free Interval, *HR* Hazard Ratio, *CI* Confidence Intervals

**Fig. 5:**
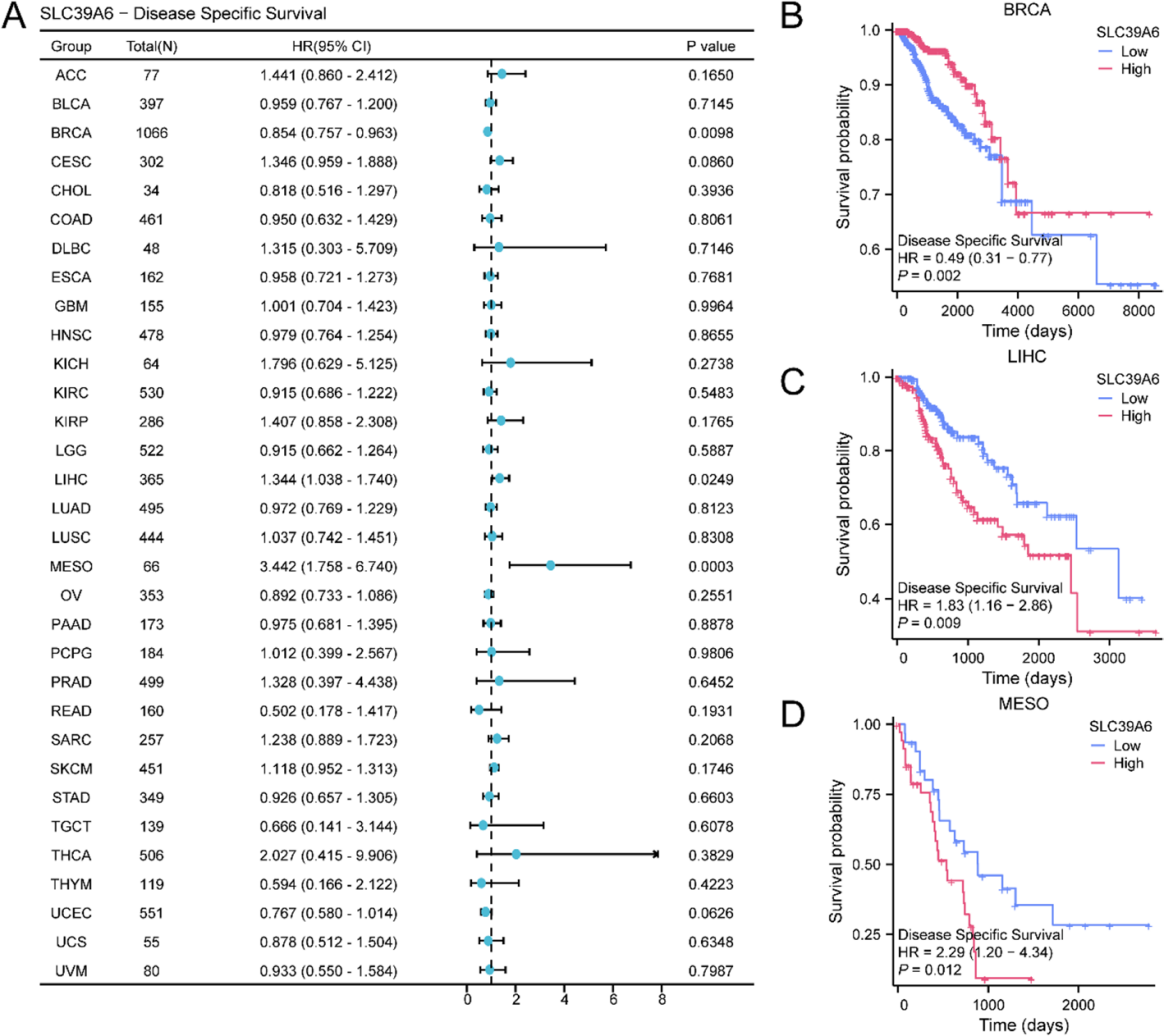
Association between SLC39A6 expression and Disease Specific Survival (DSS). **A** Forest plot of single-gene Cox analysis results of SLC39A6 disease-specific survival (DSS) in cancer samples from TCGA dataset. **B**-**D** Kaplan–meier (KM) curves of SLC39A6 in breast invasive carcinoma (BRCA) (**B**), hepatocellular carcinoma (LIHC) (**C**), and mesothelioma (MESO) (**D**) in cancer samples from TCGA dataset. TCGA, The Cancer Genome Atlas; ACC, Adrenocortical carcinoma; BLCA, Bladder urothelial carcinoma; BRCA, Breast invasive carcinoma; CESC, Cervical endocervical adenocarcinoma and squamous cell carcinoma; CHOL, Cholangiocarcinoma; COAD, Colon adenocarcinoma; DLBC, Lymphoid neoplasm diffuse large b-cell lymphoma; ESCA, Esophageal carcinoma; GBM, Glioblastoma multiforme; HNSC, Head and neck squamous cell carcinoma; KICH, Kidney chromophobe; KIRC, Kidney renal clear cell carcinoma; KIRP, Kidney renal papillary cell carcinoma; LAML, Acute myeloid leukemia; LGG, Brain lower grade glioma; LIHC, Liver hepatocellular carcinoma; LUAD, Lung adenocarcinoma; LUSC, Lung squamous cell carcinoma; MESO, Mesothelioma; OV, Ovarian serous cystadenocarcinoma; PAAD, Prostate adenocarcinoma; PCPG, Pheochromocytoma and paraganglioma; PRAD, Pancreatic adenocarcinoma; READ, Rectum adenocarcinoma; SARC, Sarcoma; SKCM, Skin cutaneous melanoma; STAD, Stomach adenocarcinoma; TGCT, Testicular germ cell tumors; THCA, Thyroid carcinoma; THYM, Thymoma; UCEC, Uterine corpus endometrial carcinoma; UCS, Uterine carcinosarcoma; UVM, Uveal melanoma; DSS, Disease Specific Survival; HR, Hazard Ratio; CI, Confidence Intervals; KM, Kaplan–Meier. The SLC39A6 Low expression group (Low) is blue, and the SLC39A6 High expression group (High) is pink

Results of single-gene Cox analysis of SLC39A6 disease-specific survival (DSS) showed that SLC39A6 was significant in single-gene Cox models of breast invasive carcinoma (BRCA), hepatocellular carcinoma (LIHC), and mesothelioma (MESO) (*p* value < 0.05). Kaplan–meier (KM) curves of SLC39A6 in DSS were also significantly different among the three types of cancer (Fig. 5B-D).

**Fig. 6:**
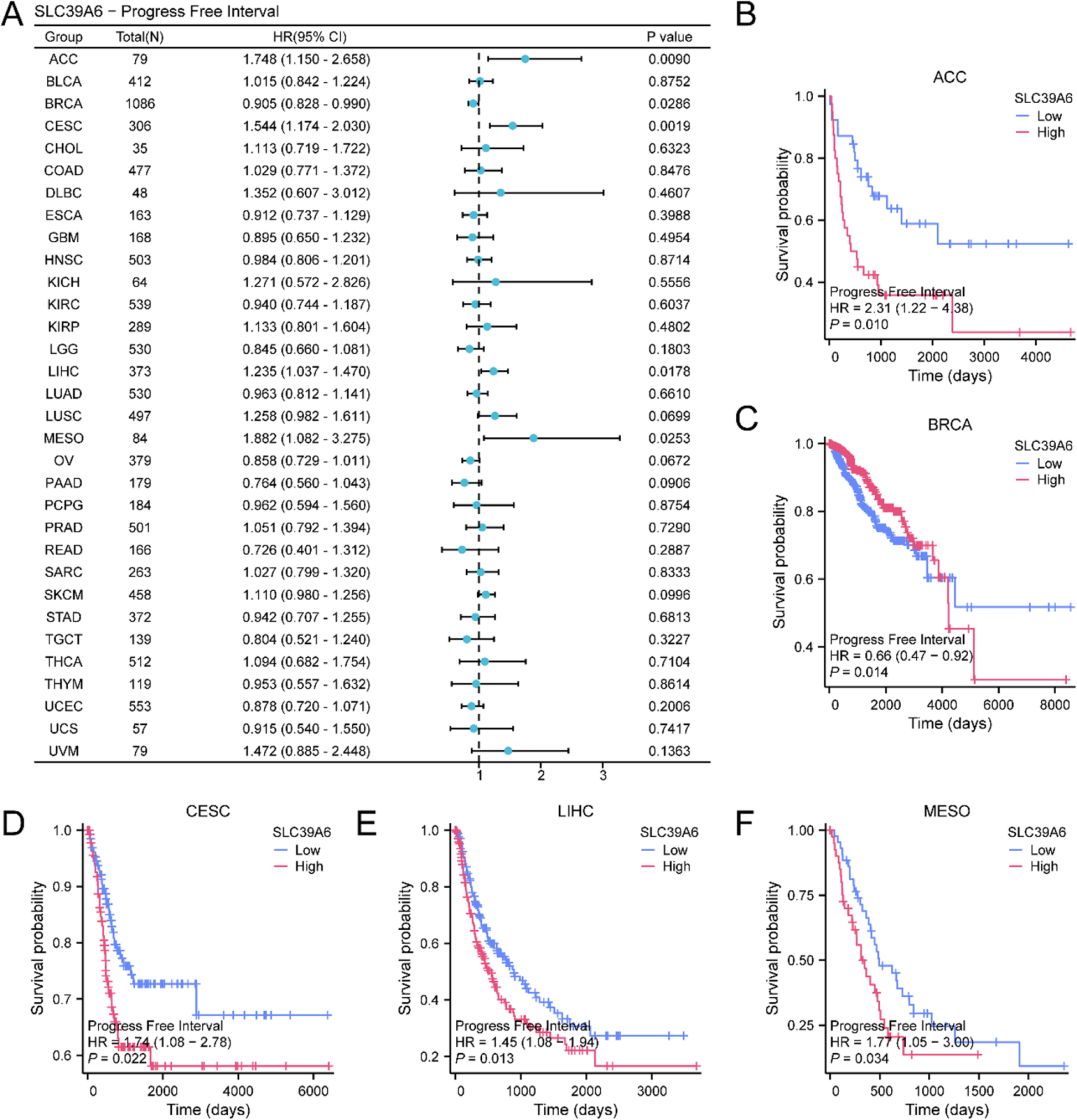
Association between SLC39A6 Expression and Progress Free Interval (PFI). **A** Forest plot of the results of single-gene Cox analysis of SLC39A6 progression-free interval (PFI) in cancer samples from TCGA dataset. **B**-**F** The expression of SLC39A6 in adrenocortical carcinoma (ACC) (**B**), invasive breast carcinoma (BRCA) (**C**), cervical squamous cell carcinoma and adenocarcinoma (CESC) (**D**), hepatocellular carcinoma (LIHC) (**E**) in cancer samples of TCGA dataset. Kaplan–meier (KM) curves in mesothelioma (MESO) (**F**). TCGA, The Cancer Genome Atlas; ACC, Adrenocortical carcinoma; BLCA, Bladder urothelial carcinoma; BRCA, Breast invasive carcinoma; CESC, Cervical endocervical adenocarcinoma and squamous cell carcinoma; CHOL, Cholangiocarcinoma; COAD, Colon adenocarcinoma; DLBC, Lymphoid neoplasm diffuse large b-cell lymphoma; ESCA, Esophageal carcinoma; GBM, Glioblastoma multiforme; HNSC, Head and neck squamous cell carcinoma; KICH, Kidney chromophobe; KIRC, Kidney renal clear cell carcinoma; KIRP, Kidney renal papillary cell carcinoma; LAML, Acute myeloid leukemia; LGG, Brain lower grade glioma; LIHC, Liver hepatocellular carcinoma; LUAD, Lung adenocarcinoma; LUSC, Lung squamous cell carcinoma; MESO, Mesothelioma; OV, Ovarian serous cystadenocarcinoma; PAAD, Prostate adenocarcinoma; PCPG, Pheochromocytoma and paraganglioma; PRAD, Pancreatic adenocarcinoma; READ, Rectum adenocarcinoma; SARC, Sarcoma; SKCM, Skin cutaneous melanoma; STAD, Stomach adenocarcinoma; TGCT, Testicular germ cell tumors; THCA, Thyroid carcinoma; THYM, Thymoma; UCEC, Uterine corpus endometrial carcinoma; UCS, Uterine carcinosarcoma; UVM, Uveal melanoma; PFI, Progress Free Interval; HR, Hazard Ratio; CI, Confidence Intervals; KM, Kaplan–Meier. The SLC39A6 Low expression group (Low) is blue, and the SLC39A6 High expression group (High) is pink

Results of single-gene Cox analysis of SLC39A6 progression-free interval (PFI) showed (Fig. 6A) that SLC39A6 was significantly associated with adrenal cortical carcinoma (ACC), invasive breast carcinoma (BRCA), cervical squamous and adenocarcinoma (CESC), hepatocellular carcinoma (LIHC). In the single-gene Cox model of mesothelioma (MESO), it was significant (*p* < 0.05). The Kaplan–Meier (KM) curves of progression-free interval (PFI) of SLC39A6 were also significantly different among the five cancers (Fig. 6B-F).

Kaplan–meier (KM) curves (OS, DSS, PFI) of SLC39A6 were significant in hepatocellular carcinoma (LIHC) and mesothelioma (MESO) (*p* < 0.05).

### Pan-cancer ssGSEA immune infiltration analysis

**Fig. 7:**
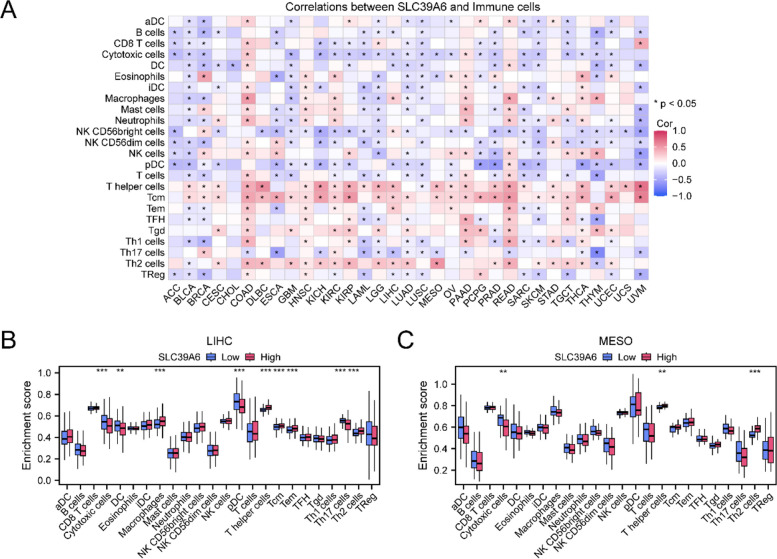
Infiltration Analysis by ssGSEA Algorithm. **A** Heat map of correlation between SLC39A6 expression and immune cell infiltration abundance in TCGA dataset samples. **B**-**c** Group comparison plots of immune cell infiltration abundance in hepatocellular carcinoma (LIHC) (**B**), mesothelioma (MESO) (**C**) with high and low SLC39A6 expression groups. TCGA, The Cancer Genome Atlas; ACC, Adrenocortical carcinoma; BLCA, Bladder urothelial carcinoma; BRCA, Breast invasive carcinoma; CESC, Cervical endocervical adenocarcinoma and squamous cell carcinoma; CHOL, Cholangiocarcinoma; COAD, Colon adenocarcinoma; DLBC, Lymphoid neoplasm diffuse large b-cell lymphoma; ESCA, Esophageal carcinoma; GBM, Glioblastoma multiforme; HNSC, Head and neck squamous cell carcinoma; KICH, Kidney chromophobe; KIRC, Kidney renal clear cell carcinoma; KIRP, Kidney renal papillary cell carcinoma; LAML, Acute myeloid leukemia; LGG, Brain lower grade glioma; LIHC, Liver hepatocellular carcinoma; LUAD, Lung adenocarcinoma; LUSC, Lung squamous cell carcinoma; MESO, Mesothelioma; OV, Ovarian serous cystadenocarcinoma; PAAD, Prostate adenocarcinoma; PCPG, Pheochromocytoma and paraganglioma; PRAD, Pancreatic adenocarcinoma; READ, Rectum adenocarcinoma; SARC, Sarcoma; SKCM, Skin cutaneous melanoma; STAD, Stomach adenocarcinoma; TGCT, Testicular germ cell tumors; THCA, Thyroid carcinoma; THYM, Thymoma; UCEC, Uterine corpus endometrial carcinoma; UCS, Uterine carcinosarcoma; UVM, Uveal melanoma; ssGSEA, single-sample Gene-Set Enrichment Analysis. * represents *p* value < 0.05, statistically significant; ** represents *p* value < 0.01, highly statistically significant; *** represents *p* value < 0.001 and highly statistically significant. In the group comparison plot, red represents the SLC39A6 High expression (High) group and blue represents the SLC39A6 Low expression (Low) group. In the correlation heat map, blue represents negative correlation, pink represents positive correlation, and the depth of color represents the strength of correlation

ssGSEA algorithm was used to calculate the infiltration abundance of 24 immune cells in 33 TCGA cancer types. According to the results of immune infiltration analysis, the heat map of the correlation between the immune infiltration abundance of 24 immune cells and SLC39A6 gene expression in 33 TCGA cancer types was plotted (Fig. 7A).

Then, the grouping comparison of immune cell infiltration abundance between high and low SLC39A6 expression groups in key cancer types hepatocellular carcinoma (LIHC), mesothelioma (MESO) (Fig. 7B-C), hepatocellular carcinoma (LIHC) (Fig. 7B) showed that the 9 immune cells, Nine kinds of immune cells, including Cytotoxic cells, DC, Macrophages, pDC, T helper cells, Tcm, Tem, Th17 cells, and Th2 cells, were statistically significant between the high and low expression groups of SLC39A6 (*p* < 0.05). The group comparison diagram of mesothelioma (MESO) (Fig. 7C) showed that three immune cells, including Cytotoxic cells, T helper cells and Th2 cells, had statistically significant differences between the high and low expression groups of SLC39A6 (*p* value < 0.05).

### Correlation analysis of pan-cancer immune checkpoint gene (ICG) and mismatch repair (MMR) genes

**Fig. 8:**
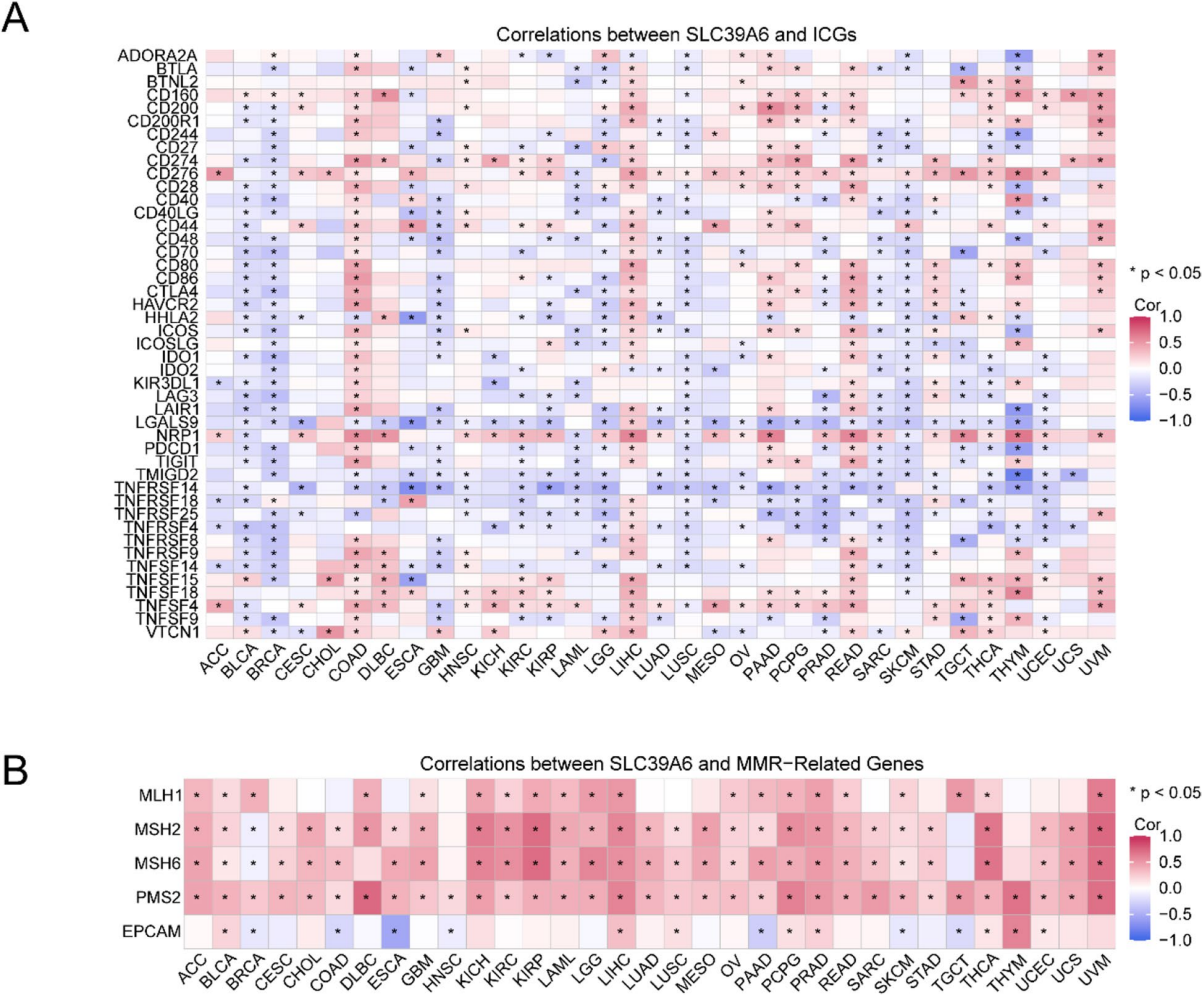
Pan-Cancer ICG, MMR Correlation Analysis of SLC39A6. **A **Heat map of pan-cancer immune checkpoint gene (ICG) correlation of SLC39A6 gene. 33 TCGA cancer types are plotted on the abscissa, and 45 immune checkpoint genes (ICG) are plotted on the ordinate. **B** Heat map of pan-cancer MMR gene association of SLC39A6 gene. 33 TCGA cancer types are plotted on the abscissa, and 5 MMR genes are plotted on the ordinate. ICG, Immune Checkpoint Genes; MMR, Mismatch Repair; TCGA, The Cancer Genome Atlas; ACC, Adrenocortical carcinoma; BLCA, Bladder urothelial carcinoma; BRCA, Breast invasive carcinoma; CESC, Cervical endocervical adenocarcinoma and squamous cell carcinoma; CHOL, Cholangiocarcinoma; COAD, Colon adenocarcinoma; DLBC, Lymphoid neoplasm diffuse large b-cell lymphoma; ESCA, Esophageal carcinoma; GBM, Glioblastoma multiforme; HNSC, Head and neck squamous cell carcinoma; KICH, Kidney chromophobe; KIRC, Kidney renal clear cell carcinoma; KIRP, Kidney renal papillary cell carcinoma; LAML, Acute myeloid leukemia; LGG, Brain lower grade glioma; LIHC, Liver hepatocellular carcinoma; LUAD, Lung adenocarcinoma; LUSC, Lung squamous cell carcinoma; MESO, Mesothelioma; OV, Ovarian serous cystadenocarcinoma; PAAD, Prostate adenocarcinoma; PCPG, Pheochromocytoma and paraganglioma; PRAD, Pancreatic adenocarcinoma; READ, Rectum adenocarcinoma; SARC, Sarcoma; SKCM, Skin cutaneous melanoma; STAD, Stomach adenocarcinoma; TGCT, Testicular germ cell tumors; THCA, Thyroid carcinoma; THYM, Thymoma; UCEC, Uterine corpus endometrial carcinoma; UCS, Uterine carcinosarcoma; UVM, Uveal melanoma. In the correlation heat map, red represents positive correlation, blue represents negative correlation, and the depth of color represents the strength of the correlation. * represents a *p* value < 0.05, which is statistically significant

In PubMed website (https://pubmed.ncbi.nlm.nih.gov/) has been published in the literature got 47 (ICG) on immune checkpoint gene, with the TCGA extensive cancer gene expression profile contained in the intersection, got 45 genes (ICG) on immune checkpoints. The correlation between the expression of 45 immune checkpoint genes (ICG) and SLC39A6 gene expression in 33 TCGA cancer types was calculated, and the correlation heat map (Fig. 8A) was drawn. The results showed that SLC39A6 was negatively correlated with the immune checkpoint gene (ICG) in most cancer types. Then, the correlation between the expression of 5 MMR genes and SLC39A6 gene in 33 TCGA cancer types was calculated and the correlation heat map was plotted (Fig. 8B). The results showed that MLH1, MSH2, MSH6, PMS2 were mainly positively correlated with SLC39A6 gene expression. The correlation between the expression of EPCAM and SLC39A6 was similar.We supplement the complete list of 45 immune checkpoint genes (ICGs) and 5 mismatch repair (MMR) genes (see Supplement 1–3 for details).

### Genetic variant analysis (TMB/MSI)

**Fig. 9:**
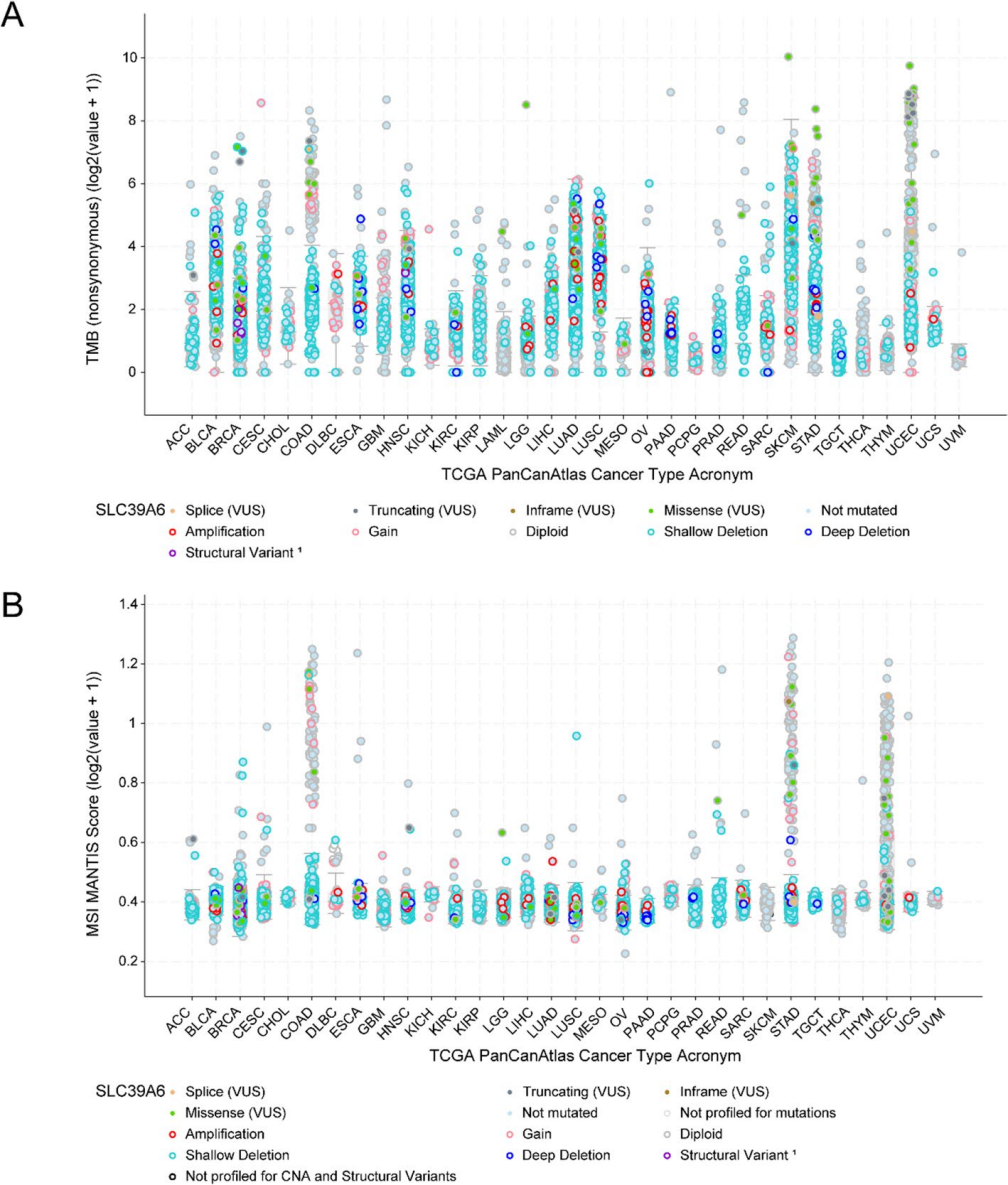
Pan-cancer TMB and MSI analysis of SLC39A6. **A** Dot plot of tumor mutation burden (TMB) of SLC39A6 gene in 33 TCGA cancer types. **B** Microsatellite instability (MSI) dot plot of SLC39A6 gene in 33 TCGA cancer types. Tumor mutation burden (TMB) or microsatellite instability (MSI) of SLC39A6 gene is plotted on the abscissor for 33 TCGA cancer types and on the ordinate. TCGA, The Cancer Genome Atlas; ACC, Adrenocortical carcinoma; BLCA, Bladder urothelial carcinoma; BRCA, Breast invasive carcinoma; CESC, Cervical endocervical adenocarcinoma and squamous cell carcinoma; CHOL, Cholangiocarcinoma; COAD, Colon adenocarcinoma; DLBC, Lymphoid neoplasm diffuse large b-cell lymphoma; ESCA, Esophageal carcinoma; GBM, Glioblastoma multiforme; HNSC, Head and neck squamous cell carcinoma; KICH, Kidney chromophobe; KIRC, Kidney renal clear cell carcinoma; KIRP, Kidney renal papillary cell carcinoma; LAML, Acute myeloid leukemia; LGG, Brain lower grade glioma; LIHC, Liver hepatocellular carcinoma; LUAD, Lung adenocarcinoma; LUSC, Lung squamous cell carcinoma; MESO, Mesothelioma; OV, Ovarian serous cystadenocarcinoma; PAAD, Prostate adenocarcinoma; PCPG, Pheochromocytoma and paraganglioma; PRAD, Pancreatic adenocarcinoma; READ, Rectum adenocarcinoma; SARC, Sarcoma; SKCM, Skin cutaneous melanoma; STAD, Stomach adenocarcinoma; TGCT, Testicular germ cell tumors; THCA, Thyroid carcinoma; THYM, Thymoma; UCEC, Uterine corpus endometrial carcinoma; UCS, Uterine carcinosarcoma; UVM, Uveal melanoma; TMB, Tumor Mutation Burden; MSI, Microsatellite Instability

Tumor mutation burden (TMB) (Fig. 9A) and microsatellite instability (MSI) (Fig. 9B) of SLC39A6 in 33 TCGA cancer types were obtained from cBioPortal database. We have also performed relevant analyses using the cBioPortal database and further investigated the associated protein alterations (as shown in Supplementary Fig. 1–2). In this study, t-SNE (t-distributed stochastic neighbor embedding) dimensionality reduction was used for visual analysis of single-cell transcriptome data. The expression level of SLC39A6 showed a significantly heterogeneous distribution pattern among different subpopulations (as shown in Supplementary Fig. 3).

### PPI interaction network and Gene ontology (GO) enrichment analysis

**Fig. 10:**
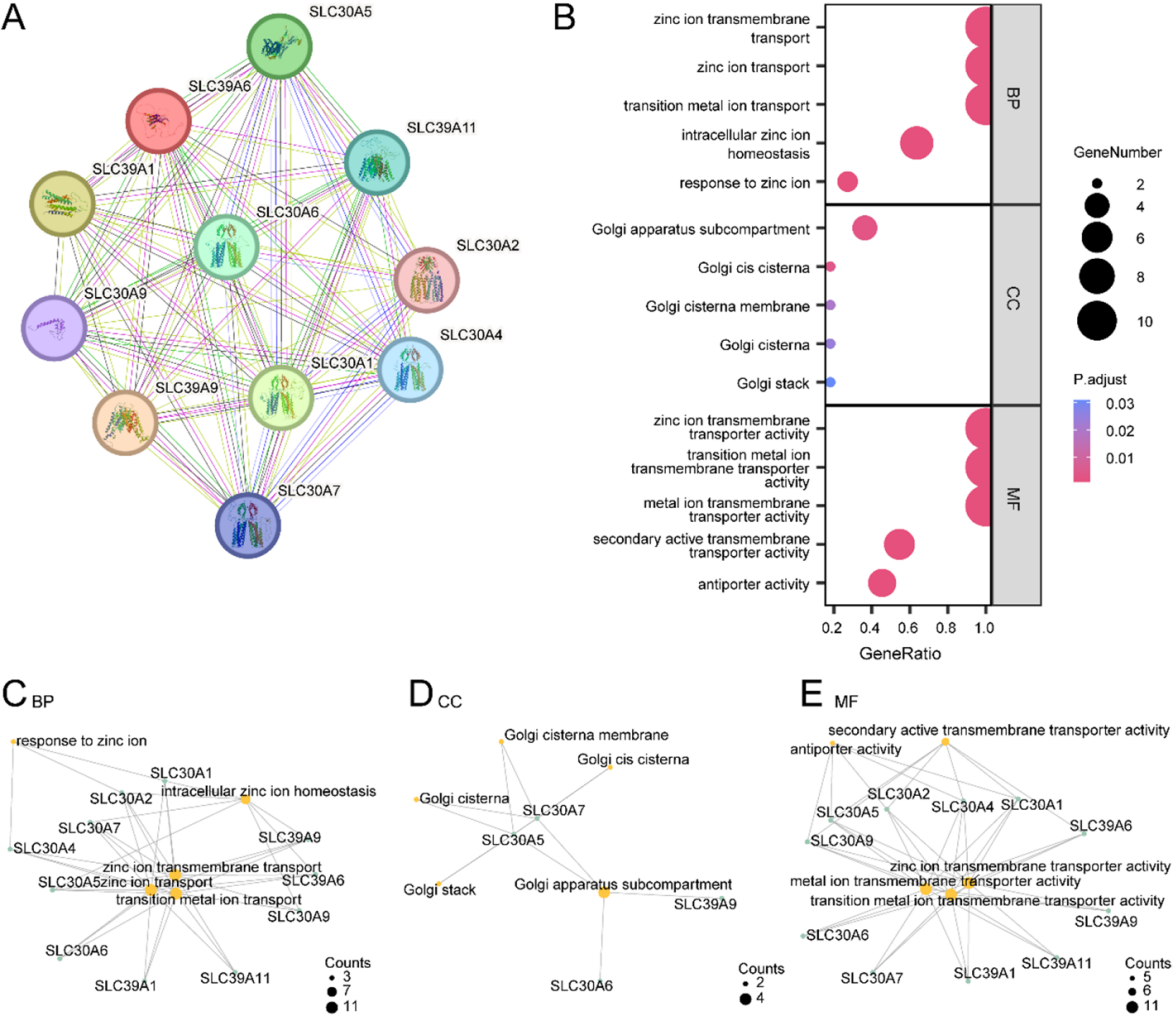
PPI Network and GO Enrichment Analysis of SLC39A6. **A** PPI interaction network of SLC39A6 gene calculated from STRING database. **B** Bubble plot of gene ontology (GO) enrichment analysis results of SLC39A6 gene and Interacting Genes: biological process (BP), cellular component (CC), molecular function (MF). The abscissa is the GO terms. **C**-**E** The gene ontology (GO) enrichment analysis results of SLC39A6 gene and Interacting Genes show: BP (**C**), CC (**D**), MF (**E**). Yellow nodes represent entries, green nodes represent molecules, and lines represent the relationship between entries and molecules. PPI, Protein–Protein Interaction; GO, Gene Ontology; BP, Biological Process; CC, Cellular Component; MF, Molecular Function. The bubble size in the bubble plot represents the number of genes, and the color of the bubble represents the size of the adj. *P*-value, the reder the color, the smaller the adj. *P*-value, and the bluer the color, the larger the adj. *P*-value. The screening criteria for gene ontology (GO) enrichment analysis were adj.p < 0.05 and FDR value (q value) < 0.25, and the *p* value correction method was Benjamini-Hochberg (BH)

Firstly, protein–protein interaction analysis was performed, and the protein–protein interaction Network (PPI Network) of SLC39A6 was constructed using STRING database (Fig. 10A). The protein–protein interaction Network (PPI Network) results showed that 10 genes were related to SLC39A6, which were: SLC30A1, SLC30A2, SLC30A4, SLC30A5, SLC30A6, SLC30A7, SLC30A9, SLC39A1, SLC39A11, SLC39A9, which were recorded as the Interacting Genes of SLC39A6.

Gene ontology (GO) enrichment analysis was used to further explore the relationship between biological process (BP), cellular component (CC), and molecular function (MF) of SLC39A6 gene and Interacting Genes. SLC39A6 gene and Interacting Genes were used for gene ontology (GO) enrichment analysis, and the specific results are shown in Table 2. The results showed that SLC39A6 gene and Interacting Genes were mainly enriched in zinc ion transmembrane transport, zinc ion transport, and zinc ion transport. transition metal ion transport, intracellular zinc ion homeostasis, response to zinc ion and other biological processes (BP); Golgi apparatus subcompartment, Golgi cis cisterna, Golgi cisterna membrane, Golgi cisterna, Golgi stack; zinc ion transmembrane transporter activity, transition metal ion transmembrane transporter activity, metal ion transmembrane transporter activity, secondary active transmembrane transporter activity, antiporter activity and other molecular functions (MF). The results of Gene ontology (GO) enrichment analysis were visualized by bubble plots (Fig. 10B).

**Table 2:** Results of GO enrichment analysis for SLC39A6 and interacting genes

ONTOLOGY	ID	Description	GeneRatio	BgRatio	*p*value	p.adjust	qvalue
BP	GO:0071577	zinc ion transmembrane transport	11/11	27/18870	4.83E-33	4.54E-31	2.97E-31
BP	GO:0006829	zinc ion transport	11/11	28/18870	7.96E-33	4.54E-31	2.97E-31
BP	GO:0000041	transition metal ion transport	11/11	100/18870	5.25E-26	1.99E-24	1.31E-24
BP	GO:0006882	intracellular zinc ion homeostasis	7/11	36/18870	1.62E-17	4.62E-16	3.03E-16
BP	GO:0010043	response to zinc ion	3/11	51/18870	3.02E-06	6.89E-05	4.52E-05
CC	GO:0098791	Golgi apparatus subcompartment	4/11	382/19886	3.98E-05	1.99E-03	1.13E-03
CC	GO:0000137	Golgi cis cisterna	2/11	31/19886	1.28E-04	3.21E-03	1.82E-03
CC	GO:0032580	Golgi cisterna membrane	2/11	93/19886	1.16E-03	1.93E-02	1.10E-02
CC	GO:0031985	Golgi cisterna	2/11	118/19886	1.85E-03	2.32E-02	1.32E-02
CC	GO:0005795	Golgi stack	2/11	151/19886	3.01E-03	3.01E-02	1.71E-02
MF	GO:0005385	zinc ion transmembrane transporter activity	11/11	24/18496	1.15E-33	3.23E-32	1.09E-32
MF	GO:0046915	transition metal ion transmembrane transporter activity	11/11	42/18496	1.98E-30	2.77E-29	9.37E-30
MF	GO:0046873	metal ion transmembrane transporter activity	11/11	441/18496	1.25E-18	1.17E-17	3.95E-18
MF	GO:0015291	secondary active transmembrane transporter activity	6/11	286/18496	5.62E-09	3.93E-08	1.33E-08
MF	GO:0015297	antiporter activity	5/11	135/18496	8.58E-09	4.80E-08	1.63E-08

*GO* Gene Ontology, *BP* Biological Process, *MF* Molecular Function

**Fig. 11:**
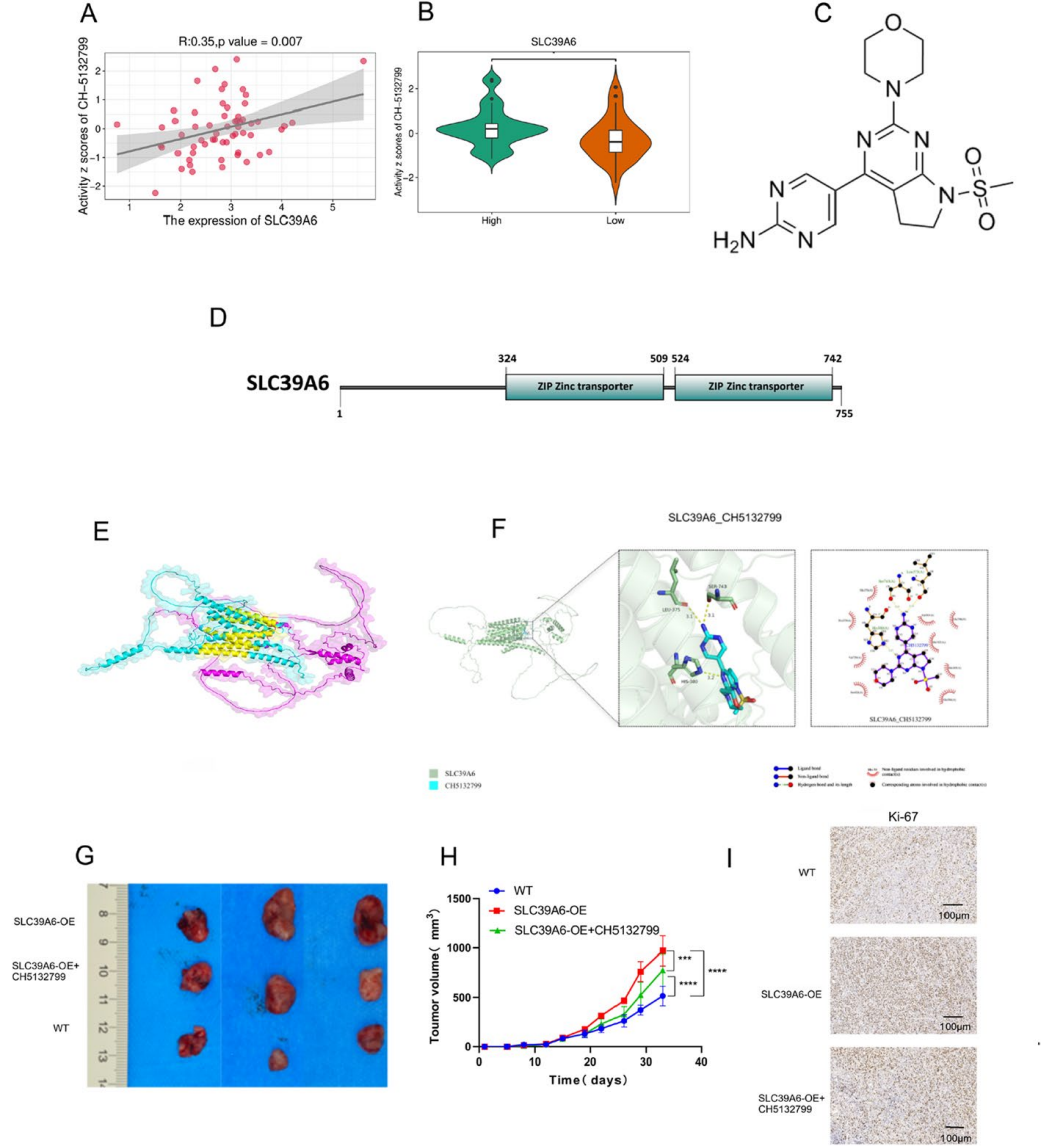
Drug sensitivity analysis of SLC39A6 and antitumor efficacy of CH5132799 in A549 xenograft model in vivo. **B** Correlation between SLC39A6 expression and CH5132799 IC50. **C**-**F** Structure and result of molecular docking simulations. **G** Representative images of tumors at 33 days after inoculation using A549 cells treated with SCL39A6-vector, SCL39A6-OE, SCL39A6-OE and CH5132799 (12.5 mg/kg once a day). **H** Tumor growth curves of the mouse xenograft study. **I** IHC analysis of Ki67 protein expression were performed using tumor sections of A549 mouse xenografts treated as indicated above. Magnification, 400 × ; scale bar, 100 µm. WT: wild type,***p < 0.001,****p < 0.0001

Meanwhile, the network maps of biological process (BP), cellular component (CC) and molecular function (MF) were drawn according to Gene ontology (GO) enrichment analysis (Fig. 10C-E). The lines show the corresponding molecules and the annotations of the corresponding entries, and the larger the nodes, the more molecules the entries contain Fig. 11.

### Drug sensitivity analysis of SLC39A6

Increased drug sensitivity is critical to prevent cancer cells from developing resistance to treatment. To explore this further, we performed a correlation analysis between drug sensitivity and SLC39A6 expression levels using data from the CellMiner database. Our analysis revealed significant positive correlations between SLC39A6 and drug sensitivity to CH5132799, of which is a PI3K inhibitor (Fig. 11 A-B). Moreover, molecular docking results revealed that the binding energy of compound CH5132799 to the SLC39A6 protein was −7.6 kcal/mol, indicating its strong inhibitory potential against SLC39A6 (Fig. 11 C-F). Meanwhile, we conducted in vivo validation in animal models. The results demonstrated that CH5132799 administration significantly inhibited tumor proliferation when SLC39A6 was highly expressed (SLC39A6-OE + CH5132799 210.0 mm^3^ ± 83.14 mm^3^ vs. SLC39A6-OE 282.8 mm^3^ ± 109.8 mm^3^, *p* < 0.001; and vs. WT 158.5 mm^3^ ± 55.69 mm^3^, *p* < 0.0001 (Fig. 11 G-H)). Immunohistochemical analysis revealed that Ki67 expression in tumor tissues was consistent with these findings (Fig. 11 I). Individual tumor growth curves are provided in in Supplementary Fig. 4. H-score of Ki67 expression are provided in in Supplementary Table 1.

## Discussion

Despite decades of progress, common solid malignancies continue to impose an unacceptable burden on global public health, and current therapeutic modalities still fall short of providing durable clinical benefits. By integrating pan-cancer, multi-omics analyses, we delineated a central oncogenic role for the zinc transporter SLC39A6 and identified it as a tractable target for precision oncology. We report several principal findings. First, SLC39A6 is markedly dysregulated across more than ten cancer types and independently predicts shortened overall survival (OS), disease-specific survival (DSS), and progression-free interval (PFI). Second, we revealed that sensitivity to the PI3Kα-selective inhibitor CH5132799 increases with SLC39A6 expression; in vivo studies confirmed that CH5132799 effectively suppresses SLC39A6-driven tumor cell proliferation. These results provide a robust foundation for translational development.

SLC39A6 is markedly up-regulated in hepatocellular carcinoma (HCC) tissues, and its expression increases progressively with higher TNM stages, enabling discrimination between early- and advanced-stage HCC [27]. A meta-analysis of nine GEO datasets revealed that SLC39A6 mRNA is significantly elevated in early-stage tumors [28]. In ER-positive breast cancer cases, high SLC39A6 protein expression is associated with longer disease-free survival, supporting its utility for molecular subtyping [29].

Univariate Cox modeling indicated that elevated SLC39A6 expression is significantly associated with poorer overall survival (OS), disease-specific survival (DSS), and progression-free interval (PFI), particularly in cervical squamous cell carcinoma (CESC), hepatocellular carcinoma (LIHC), and mesothelioma (MESO). This establishes its role as both a prognostic biomarker and a therapeutic target. Importantly, this association is context-dependent. In hormone-receptor-positive breast cancer, higher SLC39A6 levels surprisingly indicate a favorable prognosis, especially in node-negative or luminal A subtypes, whereas no prognostic significance is seen in basal-like or HER2-positive tumors [29]. These findings underscore the importance of molecular stratification prior to clinical application. In CESC and MESO, elevated SLC39A6 expression consistently signifies a poor prognosis irrespective of FIGO or TNM stage, suggesting its value as a stage-independent risk factor. In summary, the prognostic impact of SLC39A6 varies by cancer type and subtype, and it can exhibit a "double-edged sword" effect in hormone-dependent tumors. Moving forward, incorporating SLC39A6 into multi-gene, multi-modal prognostic scores and utilizing SLC39A6 immunohistochemistry (IHC) or quantitative polymerase chain reaction (qPCR) in multidisciplinary tumor boards will facilitate precise patient stratification before clinical implementation.

Immune-microenvironment analyses have revealed that SLC39A6 remodels tumor immunity in liver hepatocellular carcinoma (LIHC) and malignant mesothelioma (MESO). Single-sample gene set enrichment analysis (ssGSEA) demonstrated significant skewing of nine immune subsets—including cytotoxic T cells, dendritic cells, and macrophages—between SLC39A6-high and SLC39A6-low tumors. Spatial transcriptomic mapping in colorectal cancer showed inverse localization of SLC39A6 with CD8⁺ T cells and dendritic cells, but co-localization with M2-polarized macrophages. Mechanistically, SLC39A6 activates the Toll-like receptor and IL-17 signaling axes, triggering the release of CXCL8 and CCL2, which recruits M2 macrophages and dampens cytotoxic immunity [28]. In LIHC, SLC39A6-mediated zinc influx phosphorylates STAT3 and transcriptionally up-regulates PD-L1 and VEGF-A, thereby impairing dendritic-cell maturation and macrophage polarization. Knockdown of SLC39A6 in HepG2 and Hep3B cells restored CD8⁺ T-cell cytotoxicity, reduced PD-L1 by 45%, and synergized with anti-PD-1 therapy to shrink tumors by 65% in murine models [27]. Similarly, the SLC39A6-directed antibody–drug conjugate (ADC) BRY812 reduced M2 infiltration and restored CD8⁺ T-cell activity in patient-derived colorectal organoids; combination with anti-CTLA-4 improved objective response rates from 23 to 58% [28]. A pan-cancer immunogenomic cohort (*n* = 1,054) confirmed that SLC39A6-high tumors exhibit reduced CD8⁺ T-cell infiltration, lower tumor mutational burden, and diminished sensitivity to PD-1 blockade, suggesting that this immune-regulatory axis is conserved across multiple histologies [30].

Protein–protein interaction network analysis has identified ten proteins significantly associated with SLC39A6, including multiple members of the zinc transporter family. Collectively, these proteins participate in zinc-ion transmembrane transport and other essential biological processes. This interaction network highlights the critical role of metal-ion homeostasis and suggests that these transporters may cooperatively modulate cell proliferation and apoptosis pathways, thereby influencing tumor progression.

Beyond immunity, the disruption of the ZIP/ZnT/metallothionein axis leads to increased intracellular zinc levels, which activates the PI3K-AKT, MAPK/ERK, and STAT3 cascades. These pathways drive cell-cycle progression, epithelial-mesenchymal transition (EMT), and the evasion of apoptosis [4, 27, 28]. In models of breast and liver cancer, zinc influx mediated by SLC39A6 phosphorylates AKT, inactivates GSK-3β, and stabilizes Snail, thereby promoting metastasis. Iron, copper, and manganese exert similar effects through transporters such as ZIP8/SLC30A/DMT1/ATP7A, highlighting the broader role of metal-ion homeostasis in oncogenesis [31]. Targeting this circuitry is clinically viable: the SLC39A6-directed antibody–drug conjugate (ADC) ladiratuzumab vedotin is currently in phase I/II trials, and ZnO nanoparticles utilize NCOA4-mediated ferritinophagy to induce ferroptosis, offering a versatile drug-delivery platform [28, 32–35].

Correlative analyses have revealed that SLC39A6 inversely correlates with immune-checkpoint gene (ICG) expression, while positively correlating with the DNA-mismatch-repair genes MLH1, MSH2, MSH6, and PMS2. To our knowledge, this is the first report linking SLC39A6 to ICG/MMR networks. Low ICG expression indicates an immune-cold microenvironment and predicts a poor response to PD-1/PD-L1 blockade, whereas high MMR gene expression signifies microsatellite stability and chemoresistance to DNA-damaging agents. Integrating SLC39A6 with MMR status may therefore refine risk stratification and guide combination regimens—for instance, CH5132799 plus platinum-free immunotherapy in SLC39A6-high/MMR-intact tumors [30].

High expression of SLC39A6 sensitizes tumors to CH5132799, a selective PI3Kα inhibitor that effectively suppresses cell proliferation driven by SLC39A6. This observation is consistent with the seminal report by Tanaka, who first demonstrated potent in vitro and in vivo antitumor activity of CH5132799 against PI3K-hyperactivated tumor cells [36]. Building on this foundation, we are the first to advance SLC39A6 from a prognostic indicator to an actionable companion-diagnostic biomarker for CH5132799. Clinically, high SLC39A6 expression can be used to identify patients most likely to benefit from this agent. Phase II basket trials are planned in cervical squamous cell carcinoma (CESC), hepatocellular carcinoma (LIHC), and mesothelioma (MESO). Given CH5132799’s exquisite selectivity for SLC39A6-high tumors, combination strategies with PD-1 inhibitors or zinc chelators could overcome immune-cold phenotypes and potentiate metal-ion-induced ferroptosis.We systematically reviewed and compared existing SLC39A6-targeted strategies (ADC, zinc chelation, RNAi) and summarized their inherent limitations, such as poor specificity, low efficacy, and high toxicity.Different from previous single-cancer or single-strategy studies, our research conducts a comprehensive pan-cancer analysis of SLC39A6, identifies it as a companion diagnostic biomarker for the PI3Kα inhibitor CH5132799, clarifies its related molecular mechanisms, and thus fills the gap in SLC39A6-targeted precision oncology.

Limitations encompass the retrospective nature of publicly available datasets, uneven sample sizes, and the lack of comprehensive confounder adjustment. Failure to integrate co-expression and interaction data is a limitation of this study. In subsequent research, we plan to combine TCGA and other public databases to construct a multi-layer network integrating expression correlation and protein interaction evidence, so as to further analyze the functional modular characteristics of oxidative stress-related genes. Further mechanistic studies and prospective validation are necessary to confirm the function of SLC39A6 and to assess its full potential as a therapeutic target and predictive biomarker.

## Conclusion

In conclusion, our study determined SLC39A6 regulates the dynamics of immune infiltration and impacts prognosis in a wide range of malignancies. Besides, our results advance SLC39A6 from a prognostic indicator to an actionable companion-diagnostic biomarker for CH5132799. It emerges as a promising biomarker for prognosis, immunology, and therapy in the field of precision oncology.

## Supplementary Information

Supplementary Material 1.

## Data Availability

The original data presented in the study are included in the article materials. Further inquiries can be directed to the corresponding authors.

## Abbreviations

ACC: Adrenocortical carcinoma
BP: Biological Process
CC: Cell Component
CESC: Cervical endocervical adenocarcinoma and squamous cell carcinoma
CHOL: Cholangiocarcinoma
COAD: Colon adenocarcinoma
DLBC: Lymphoid neoplasm diffuse large B-cell lymphoma
DSS: Disease-specific survival
ESCA: Esophageal carcinoma
FPKM: Fragments Per Kilobase Million
GBM: Glioblastoma multiforme
GO: Gene Ontology
HNSC: Head and neck squamous cell carcinoma
IHC: Immunohistochemistry
ICG: Immune Checkpoint Genes
KICH: Kidney chromophobe
KIRC: Kidney renal clear cell carcinoma
KIRP: Kidney renal papillary cell carcinoma
LAML: Acute myeloid leukemia
LGG: Brain lower grade glioma
LIHC: Liver hepatocellular carcinoma
LUAD: Lung adenocarcinoma
LUSC: Lung squamous cell carcinoma
KM: Kaplan–Meier
MESO: Mesothelioma
MF: Molecular Function
MMR: Mismatch Repair
MSI: Microsatellite instability
OS: Overall survival
OV: Ovarian serous cystadenocarcinoma
PAAD: Prostate adenocarcinoma
PCPG: Pheochromocytoma and paraganglioma
PFI: Progression-free interval
PPI: Protein–Protein Interaction Network
PRAD: Pancreatic adenocarcinoma
qPCR: Quantitative polymerase chain reaction
READ: Rectum adenocarcinoma
SARC: Sarcoma
SKCM: Skin cutaneous melanoma
SLC39A6: Solute carrier family 39 member 6
ssGSEA: Single-Sample Gene-Set Enrichment Analysis
STAD: Stomach adenocarcinoma
TCGA: The Cancer Genome Atlas
TGCT: Testicular germ cell tumors
THCA: Thyroid carcinoma
THYM: Thymoma
TMB: Tumor mutational burden
UCEC: Uterine corpus endometrial carcinoma
UCS: Uterine carcinosarcoma
ZIP: Zrt-Irt-like protein

## References

[1] Sung H, Ferlay J, Siegel RL, Laversanne M, Soerjomataram I, Jemal A, et al. Global cancer statistics 2020: GLOBOCAN estimates of incidence and mortality worldwide. CA Cancer J Clin. 2021;71(3):209–49. 10.3322/caac.21660. 33538338 10.3322/caac.21660

[2] Siegel RL, Miller KD, Fuchs HE, Jemal A. Cancer statistics, 2023. CA Cancer J Clin. 2023;73(1):17–48. 10.3322/caac.21763. 36633525 10.3322/caac.21763

[3] Prasad RR, Singh CK, Meng J, Nagamuga S, Satarug S. Stage-specific differential expression of zinc transporter SLC39A6 during tumorigenesis. Mol Carcinog. 2022;61(5):454–71. 10.1002/mc.23361. 35049094 10.1002/mc.23382 PMC11952371

[4] Fukada T, Kambe T. Molecular and genetic features of zinc transporters in physiology and pathogenesis. Metallomics. 2011;3(7):662–74. 10.1039/c1mt00011j. 10.1039/c1mt00011j 21566827

[5] Saravanan R, Taylor KM, Brethour D. Zinc transporter LIV-1 (SLC39A6): a promising cell-surface target for triple-negative breast cancer. J Cell Physiol. 2022;237(12):4471–81. 10.1002/jcp.30841. 10.1002/jcp.30880 36181695

[6] Liu MY, Cui XB, Cheng XX, Zhao L, Chen W, Li X. SLC39A6 promotes aggressiveness of esophageal carcinoma by activating PI3K signaling via zinc influx. Gastroenterology. 2021;160(6):2228–40. 10.1053/j.gastro.2021.02.054.33865806

[7] Zhou H, Wang J, Li Y, Zhang Y, Wang X, Li Z. Evaluation of the prognostic value of SLC39A6 in lung adenocarcinoma using TCGA data. Aging Albany NY. 2021;13(4):5312–27. 10.18632/aging.202640.33535184

[8] Lyu G, Li D. ZNF165: a pan-cancer biomarker with prognostic and therapeutic potential. Protein Pept Lett. 2025;32(3):206–23. 10.2174/0109298665351592250106062250. 39865831 10.2174/0109298665351592250106062250

[9] Zhang Y, Zhao Z, Huang W, et al. Pan-cancer single-cell analysis revealing the heterogeneity of cancer-associated fibroblasts in skin tumors. Curr Gene Ther. 2025;25(5):793–821. 10.2174/0115665232331353240911080642. 39323331 10.2174/0115665232331353240911080642

[10] Shi Y, Shen Q, Jiang A, et al. DrugSurvPlot: a novel web-based platform harnessing drug sensitivity scores as molecular biomarkers for pan-cancer survival prognosis. Curr Gene Ther. 2025. 10.2174/0115665232412138250722020114. 10.2174/0115665232412138250722020114 40734432

[11] Jiang M, Zhu D, Zhao D, et al. Integrated analysis of clinical outcome of mesenchymal stem cell-related genes in pan-cancer. Curr Genomics. 2024;25(4):298–315. 10.2174/0113892029291247240422060811. 39156727 10.2174/0113892029291247240422060811 PMC11327807

[12] Colaprico A, Silva TC, Olsen C, Garofano L, Cava C, Garolini D, et al. TCGAbiolinks: an R/Bioconductor package for integrative analysis of TCGA data. Nucleic Acids Res. 2016;44(8):e71. 10.1093/nar/gkv1507. 26704973 10.1093/nar/gkv1507 PMC4856967

[13] Goldman MJ, Craft B, Hastie M, Repečka K, McDade F, Kamath A, et al. Visualizing and interpreting cancer genomics data via the Xena platform. Nat Biotechnol. 2020;38(6):675–8. 10.1038/s41587-020-0546-8. 32444850 10.1038/s41587-020-0546-8 PMC7386072

[14] Therneau TM. A package for survival analysis in R. R package version 3.4–0. 2022. https://CRAN.R-project.org/package=survival.

[15] Rich JT, Neely JG, Paniello RC, Voelker CC, Nussenbaum B, Wang EW. A practical guide to understanding Kaplan-Meier curves. Otolaryngol Head Neck Surg. 2010;143(3):331–6. 10.1016/j.otohns.2010.05.007. 20723767 10.1016/j.otohns.2010.05.007 PMC3932959

[16] Xiao B, Liu L, Li A, Liu J, Hu Z, Xiang D, et al. Identification and verification of immune-related gene prognostic signature based on ssGSEA for osteosarcoma. Front Oncol. 2020;10:607622. 10.3389/fonc.2020.607622. 33384961 10.3389/fonc.2020.607622 PMC7771722

[17] Xu D, Liu X, Wang Y, Li X, Zhang H, Wang J. Identification of immune subtypes and prognosis of hepatocellular carcinoma based on immune checkpoint gene expression profile. Biomed Pharmacother. 2020;126:109903. 10.1016/j.biopha.2020.109903. 32113055 10.1016/j.biopha.2020.109903

[18] Chen R, Wu W, Chen SY, Chen X, Liu C, Chen L, et al. A pan-cancer analysis reveals CLEC5A as a biomarker for cancer immunity and prognosis. Front Immunol. 2022;13:831542. 10.3389/fimmu.2022.831542. 35979347 10.3389/fimmu.2022.831542 PMC9376251

[19] de Bruijn I, Kundra R, Mastrogiacomo B, Zhang H, Jolly S, Luna A, et al. Analysis and visualization of longitudinal genomic and clinical data from the AACR Project GENIE Biopharma Collaborative in cBioPortal. Cancer Res. 2023;83(23):3861–7. 10.1158/0008-5472.CAN-23-1501. 37668528 10.1158/0008-5472.CAN-23-0816 PMC10690089

[20] Cerami E, Gao J, Dogrusoz U, Gross BE, Sumer SO, Aksoy BA, et al. The cBio cancer genomics portal: an open platform for exploring multidimensional cancer genomics data. Cancer Discov. 2012;2(5):401–4. 10.1158/2159-8290.CD-12-0095. 22588877 10.1158/2159-8290.CD-12-0095 PMC3956037

[21] Gao J, Aksoy BA, Dogrusoz U, Dresdner G, Gross B, Sumer SO, Sun Y, Jacobsen A, Sinha R, Larsson E, Cerami E, Sander C, Schultz N. Integrative analysis of complex cancer genomics and clinical profiles using the cBioPortal. Sci Signal. 2013;6(269):pl1. 10.1126/scisignal.2004088. 10.1126/scisignal.2004088 PMC4160307 23550210

[22] Szklarczyk D, Kirsch R, Koutrouli M, Nastou KC, Mehryary F, Hachilif R, et al. The STRING database in 2023: protein-protein association networks and functional enrichment analyses for any sequenced genome of interest. Nucleic Acids Res. 2023;51(D1):D638–46. 10.1093/nar/gkac1000. 36370105 10.1093/nar/gkac1000 PMC9825434

[23] Mi H, Muruganujan A, Ebert D, Huang X, Thomas PD. PANTHER version 14: more genomes, a new PANTHER GO-slim and improvements in enrichment analysis tools. Nucleic Acids Res. 2019;47(D1):D419–26. 10.1093/nar/gky1038. 30407594 10.1093/nar/gky1038 PMC6323939

[24] Yu G, Wang LG, Han Y, He QY. clusterProfiler: an R package for comparing biological themes among gene clusters. OMICS. 2012;16(5):284–7. 10.1089/omi.2011.0118. 22455463 10.1089/omi.2011.0118 PMC3339379

[25] Shankavaram UT, Varma S, Kane D, Sunshine M, Chary KK, Reinhold WC, et al. Cell Miner: a relational database and query tool for the NCI-60 cancer cell lines. BMC Genomics. 2009;10:277. 10.1186/1471-2164-10-277. 19549304 10.1186/1471-2164-10-277 PMC2709662

[26] Reinhold WC, Sunshine M, Varma S, Doroshow JH, Pommier Y. Using CellMiner 1.6 for systems pharmacology and genomic analysis of the NCI-60. Clin Cancer Res. 2015;21(17):3841–3852. 10.1158/1078-0432.CCR-15-0335. 10.1158/1078-0432.CCR-15-0335 PMC4558215 26048278

[27] Wan Z, Wang X. Role of SLC39A6 in the development and progression of liver cancer. Oncol Lett. 2022;23(3):77. 10.3892/ol.2022.13197. 35111246 10.3892/ol.2022.13197 PMC8771636

[28] Liu X, Liu W, Wu Y, Wang Y, Jiang Q, Li Y, et al. Investigation of the cytotoxic effects and mechanisms of the SLC39A6-targeting ADC drug BRY812 in CRC. Sci Rep. 2025;15(1):18275. 10.1038/s41598-025-03713-1. 40414981 10.1038/s41598-025-03713-1 PMC12104431

[29] Althobiti M, El-Sharawy KA, Joseph C, Aleskandarany M, Toss MS, Green AR, et al. Oestrogen-regulated protein SLC39A6: a biomarker of good prognosis in luminal breast cancer. Breast Cancer Res Treat. 2021;189(3):621–30. 10.1007/s10549-021-06336-y. 34453638 10.1007/s10549-021-06336-y PMC8505289

[30] Charoentong P, Finotello F, Angelova M, et al. Pan-cancer immunogenomic analyses reveal genotype–immunophenotype relationships and predictors of response to checkpoint blockade. Cell Rep. 2017;18(1):248–62. 10.1016/j.celrep.2016.12.019. 28052254 10.1016/j.celrep.2016.12.019

[31] Zhou D, Lu P, Mo X, et al. Ferroptosis and metabolic syndrome and complications: association, mechanism, and translational applications. Front Endocrinol (Lausanne). 2024;14:1248934. 10.3389/fendo.2023.1248934. 10.3389/fendo.2023.1248934 PMC10800994 38260171

[32] Sussman D, Smith LM, Anderson ME, et al. SGN-LIV1A: a novel antibody-drug conjugate targeting LIV-1 for the treatment of metastatic breast cancer. Mol Cancer Ther. 2014;13(12):2991–3000. 10.1158/1535-7163.MCT-13-0896. 25253783 10.1158/1535-7163.MCT-13-0896

[33] Cui XB, Shen YY, Jin TT, et al. SLC39A6: a potential target for diagnosis and therapy of esophageal carcinoma. J Transl Med. 2015;13:321. 10.1186/s12967-015-0681-z. 26444413 10.1186/s12967-015-0681-z PMC4595240

[34] Qin X, Zhang J, Wang B, et al. Ferritinophagy is involved in the zinc oxide nanoparticles-induced ferroptosis of vascular endothelial cells. Autophagy. 2021;17(12):4266–4285. 10.1080/15548627.2021.1911016. 10.1080/15548627.2021.1911016 PMC8726675 33843441

[35] Bartos A, Sikora J. Bioinorganic modulators of ferroptosis: a review of recent findings. Int J Mol Sci. 2023;24(4):3634. 10.3390/ijms24043634. 10.3390/ijms24043634 PMC9967694 36835045

[36] Tanaka H, Yoshida M, Tanimura H, Fujii T, Sakata K, Tachibana Y, et al. The selective class I PI3K inhibitor CH5132799 targets human cancers harboring oncogenic PIK3CA mutations. Clin Cancer Res. 2011;17(10):3272–81. 10.1158/1078-0432.CCR-10-2882. 21558396 10.1158/1078-0432.CCR-10-2882

