# Supplementary material for: SLC39A6 as a pan-cancer promising biomarker and actionable therapeutic target for CH5132799 sensitivity
Source: BMC Cancer. 2026 May 18;26:841. doi: 10.1186/s12885-026-16063-6 (PMC13366974; doi:10.1186/s12885-026-16063-6)
Supplement: Supplementary file 1 — Supplementary Material 1. [file 12885_2026_16063_MOESM1_ESM.zip › Supplementary files/Supplementary Figure.docx]

Supplementary Figure 1 Analysis of Phosphorylation Modification and Tumor Expression Characteristics of SLC39A6 Protein

(A) Schematic diagram of the protein structure and phosphorylation site distribution of SLC39A6. The horizontal axis represents the amino acid residue number, the vertical axis represents the number of references, and the dots represent the reported phosphorylation sites. (B) Box plot showing the relationship between the phosphorylation level (Z-value) of SLC39A6 at the T479 site and pathological stage in lung adenocarcinoma (CPTAC dataset). (C) Box plots showing the differences in SLC39A6 protein expression levels (Z-value) between normal tissues and primary tumors in clear cell renal cell carcinoma (left) and ovarian cancer (right) (CPTAC dataset). (D) Bar chart showing the percentage of mutated samples of SLC39A6 protein in different tumors.

Supplementary Figure2 Analysis of the frequency and types of genetic variations of SLC39A6 gene in pan-cancer.

The bar chart shows the total alteration frequency of SLC39A6 gene in different tumor types, where different colors represent different types of alterations: green for Mutation, red for Amplification, blue for Deep Deletion, purple for Structural Variant, and gray for Multiple Alterations. The "+" marks below indicate that the corresponding variation data (structural variation data, mutation data, copy number variation data) are available for that tumor type.

Supplementary Figure 3 Single-cell gene expression distribution analysis based on t-SNE.

The t-distributed stochastic neighbor embedding (t-SNE) dimensionality reduction plot shows the two-dimensional distribution of single-cell transcriptome data. The horizontal axis represents tSNE1, and the vertical axis represents tSNE2. Each dot represents a single cell, and the color gradient of the dots indicates the expression level of the target gene, with blue/light blue representing low expression and transitioning to orange/red representing high expression.

Supplementary Figure 4 Tumor Growth in Different Mouse Groups

Each line represents an individual mouse. Blue lines indicate WT group (n=3), red lines indicate SLC39A6-OE group (n=3), and green lines indicate SLC39A6-OE+CH5132799 group (n=3). Tumor volume was measured every 3-4 days over a 33-day period.
